# Adaptive Evolution of Mitochondrial Energy Metabolism Genes Associated with Increased Energy Demand in Flying Insects

**DOI:** 10.1371/journal.pone.0099120

**Published:** 2014-06-11

**Authors:** Yunxia Yang, Shixia Xu, Junxiao Xu, Yan Guo, Guang Yang

**Affiliations:** College of Life Sciences, Nanjing Normal University, Nanjing, Jiangsu Province, China; Universidade Federal do Rio de Janeiro, Brazil

## Abstract

Insects are unique among invertebrates for their ability to fly, which raises intriguing questions about how energy metabolism in insects evolved and changed along with flight. Although physiological studies indicated that energy consumption differs between flying and non-flying insects, the evolution of molecular energy metabolism mechanisms in insects remains largely unexplored. Considering that about 95% of adenosine triphosphate (ATP) is supplied by mitochondria via oxidative phosphorylation, we examined 13 mitochondrial protein-encoding genes to test whether adaptive evolution of energy metabolism-related genes occurred in insects. The analyses demonstrated that mitochondrial DNA protein-encoding genes are subject to positive selection from the last common ancestor of Pterygota, which evolved primitive flight ability. Positive selection was also found in insects with flight ability, whereas no significant sign of selection was found in flightless insects where the wings had degenerated. In addition, significant positive selection was also identified in the last common ancestor of Neoptera, which changed its flight mode from direct to indirect. Interestingly, detection of more positively selected genes in indirect flight rather than direct flight insects suggested a stronger selective pressure in insects having higher energy consumption. In conclusion, mitochondrial protein-encoding genes involved in energy metabolism were targets of adaptive evolution in response to increased energy demands that arose during the evolution of flight ability in insects.

## Introduction

Oxidative phosphorylation (OXPHOS) is the main pathway for production of adenosine triphosphate (ATP) and provides about 95% of the energy required for the basic activities of life. According to the chemiosmotic coupling hypothesis, the electron transport chain and OXPHOS are coupled by a proton gradient across the inner mitochondrial membrane [1], thus making mitochondria the main site of energy production in cells. There are five complexes in OXPHOS: NADH dehydrogenase (complex I), succinate dehydrogenase (complex II), cytochrome *bc*_1_ complex (complex III), cytochrome *c* oxidase (complex IV), and ATP synthase (complex V) [2], [3]. All of these functional complexes except for II are encoded by mitochondrial genes [4]. Previous studies confirmed that mitochondrial protein-encoding genes underwent positive selection in animals that have higher energy demands for locomotion, such as diving cetaceans, flying bats, and alpacas living at high altitudes [5]–[7], which highlights the important role that mitochondrial protein-encoding genes play in energy metabolism [8].

Insects belong to phylum Arthropoda, the most diverse group of animals in the world [9]. As part of the earliest terrestrial faunas [10], insects originated approximately 400 million years ago (the Early Silurian). During the early evolutionary stage up until the mid-Devonian, insects lacked wings [11]. The appearance of wings improved insect environmental adaptation, which allowed them to occupy various ecological niches including terrestrial, aquatic and aerial [12]. The ability to fly also enhanced the locomotion of insects for breeding, feeding, and avoiding predators. Two evolutionary locomotive mechanisms of insects have been identified [13]. The first is direct flight, where the wings are directly connected to muscles and are unfolded, which refers to the hinging mechanism of wings. The wings of the ancient insect group Paleoptera, including orders Ephemeroptera and Odonata, have this mechanism [14]. The second mechanism is indirect flight with wings having a flexion mechanism that is present in almost all extant insects of Neoptera. Indirect flight involves muscles that are attached to the thorax to drive wing movement, with the wings acting as extensions of the thoracic exoskeleton [15].

Different modes of locomotion require different levels of energy expenditure, and thus drive relevant evolution of energy metabolism-related genes. However, the molecular evolution of insect energy metabolism-related genes remains poorly understood. Mitterboeck and Adamowicz [16] tested the association between insect flight loss and molecular substitution rates, and found relaxed selective constraints at mtDNA protein-coding loci related to energy metabolism in the wingless insects. However, their study did not specially address the selective pressure on mitochondrial energy metabolism genes in flying insects. In this study, mitochondrial DNA (mtDNA) protein-encoding genes in the OXPHOS pathway were used to investigate the molecular mechanisms of energy metabolism in insects. Our aim is to test whether mitochondrial protein-encoding genes of flying and non-flying insects underwent different selective pressures, and also to evaluate whether insects with different locomotive patterns (i.e. direct vs. indirect) experienced different selective regimes.

## Materials and Methods

### Sequence Acquisition

Nucleotide sequences of 13 mtDNA protein-encoding genes were downloaded from the MitoZoa database (http://srv00.ibbe.cnr.it/mitozoa/) [17] for 77 species that represent major insect groups (Table S1). These sequences were translated into amino acid sequences using MEGA 5 [18]. Amino acid sequences for each gene were aligned by Muscle [19] using the default settings, verified by visual inspection, and used as guides to align nucleotide sequences in MEGA 5.

### Selection Test

The ratio of non-synonymous to synonymous substitutions rate (ω = *d_N_/d_S_*) in homologous protein-coding sequences is an evaluative criterion for Darwinian selection where ω = 1, ω<1 and ω>1 correspond to neutral evolution, purifying selection and positive selection, respectively [20]. The ω value was estimated by the codon-based maximum likelihood method implemented in the CodeML program of the PAML v. 4.7 package [21]. For all CodeML analyses, nucleotide sequences were translated to proteins using the genetic code table of invertebrate mitochondria (setting: icode = 4). Alignment gaps were treated as ambiguous characters (setting: cleandata = 0). All models corrected the average nucleotide frequencies at the three codon positions (setting: CodonFreq = 2). Given that the molecular phylogenetic relationship among major insect groups, such as Hymenoptera, Coleoptera, Diptera, Lepidoptera, is a matter of substantial controversy and remains unclear, a phylogeny (Figure S1) based on previous studies [22]–[24] was used as the working topology in PAML analysis.

To detect the variation of selective pressures among different insect lineages, we used free ratio model, which allows ω variation among branches, to estimate ω value on each branch. Only ω values associated with terminal branches were used in the subsequent comparative analyses of selective pressure between flying and non-flying insects.

To examine selective pressure on the last common ancestor (LCA) of Pterygota that possessed initial flight ability and the LCA of Neoptera in which the locomotion mode changed from direct flight to indirect flight, the branch model and branch site model were implemented by PAML in the present study. First, one-ratio model (M0), the most simple model, which has the same ω ratio for all branches in the phylogeny and all sites in each gene, was used to preliminarily estimate the ω value for each mitochondrial protein-encoding gene [25], [26]. Then, two-ratio models (A, 0<ω0<1, ω1≥1), which allow a background ω ratio and a different ω on the branch of interest, were useful for detecting selective pressure acting on particular lineages [26], [27]. For null hypotheses, we used the one-ratio model and two-ratio model with a fixed ω = 1 (B, 0<ω0<1, ω1 = 1) on the branch under analysis. Lastly, since positive selection can often act on a few sites and in a short period of evolutionary time, the branch-site model was introduced [28] to test positive selection on a small number of sites along pre-specified lineages. The alternative (MA, positive selection: 0<ω_0_<1, ω_1_ = 1, ω_2_≥1) and the null model (MA0, neutral evolution with ω_2_ = 1 fixed) in the branch-site test were used to detect selective pressure on each branch.

Pairwise models were compared with critical values of the Chi square distribution using likelihood ratio test (LRT) statistics evaluated by calculating twice the log-likelihood (2ΔLn). The degrees of freedom were the difference in the number of free parameters between models. The Bayes empirical Bayes method implemented in the CodeML program of PAML v. 4.7 package [21] was used to calculate the posterior probabilities that each site belongs to the site class of positive selection on the foreground lineages.

## Results

### Molecular Evolution of mtDNA Protein-encoding Genes in Insects with different Flight Abilities

The one-ratio model analyses of 13 mtDNA protein-encoding genes showed that the ω values for each gene (range from 0.009 to 0.040) were significantly less than 1 (Table 1 and Table S2), suggesting that the function of these genes are existent and they have experienced constrained selective pressure to maintain the function.

**Table 1:** Likelihood ratio tests of selective pressures on mtDNA genes between flying and non-flying insects.

Gene	Model	-lnL	2ΔlnL	P level	Parameters
atp6	one ratio	31983.802			ω_0_ = 0.026
	two ratio	31981.025	5.554	0.018	ω_0_ = 0.021 ω_1_ = 0.029
atp8	one ratio	9970.289			ω_0_ = 0.009
	two ratio	9969.737	1.104	0.293	ω_0_ = 0.023 ω_1_ = 0.008
cox1	one ratio	57994.153			ω_0_ = 0.022
	two ratio	57988.663	10.980	0.001	ω_0 = _0.019 ω_1 = _0.023
cox2	one ratio	30070.700			ω_0_ = 0.026
	two ratio	30070.260	0.880	0.348	ω_0_ = 0.024 ω_1_ = 0.027
cox3	one ratio	35894.893			ω_0_ = 0.040
	two ratio	35894.721	0.344	0.558	ω_0_ = 0.041 ω_1_ = 0.039
cyt b	one ratio	49988.410			ω_0_ = 0.035
	two ratio	49988.386	0.048	0.827	ω_0_ = 0.035 ω_1_ = 0.035
nd1	one ratio	44931.572			ω_0_ = 0.030
	two ratio	44926.870	9.405	0.002	ω_0_ = 0.025 ω_1_ = 0.033
nd2	one ratio	63315.139			ω_0_ = 0.040
	two ratio	63311.553	7.173	0.007	ω_0_ = 0.033 ω_1_ = 0.044
nd3	one ratio	18832.555			ω_0_ = 0.030
	two ratio	18830.644	3.822	0.050	ω_0_ = 0.035 ω_1_ = 0.026
nd4	one ratio	69771.825			ω_0_ = 0.033
	two ratio	69766.696	10.259	0.001	ω_0_ = 0.028 ω_1_ = 0.036
nd4l	one ratio	15956.655			ω_0_ = 0.033
	two ratio	15953.099	7.111	0.007	ω_0_ = 0.024 ω_1_ = 0.038
nd5	one ratio	90180.389			ω_0_ = 0.027
	two ratio	90168.985	22.807	0.000	ω_0_ = 0.020 ω_1_ = 0.030
nd6	one ratio	32896.124			ω_0_ = 0.025
	two ratio	32896.104	0.041	0.840	ω_0_ = 0.024 ω_1_ = 0.025

**Figure 1:**
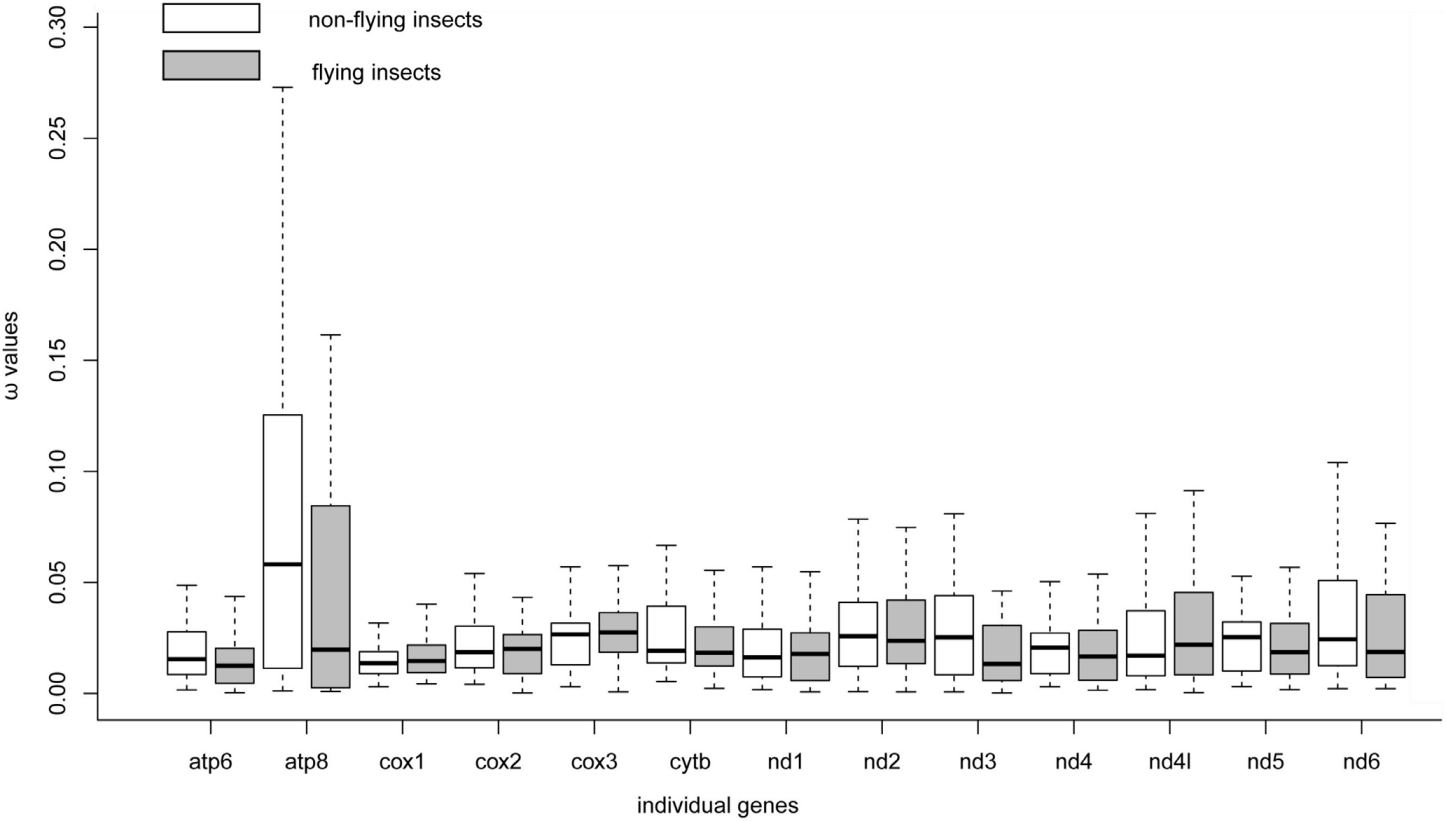
Difference in ω values of 13 protein-coding genes from flying and non-flying insects. The differences in ω value in both non-flying insects (compared on the left with white color) and flying insects (compared on the right with gray color) are shown for all mtDNA encoding genes. P values for each gene is 0.410 (atp6), 0.104 (atp8), 0.412 (cox1), 0.751 (cox2), 0.250 (cox3), 0.598 (cyt b), 0.914 (nd1), 0.962 (nd2), 0.236 (nd3), 0.528 (nd4), 0.421 (nd4l), 0.817 (nd5), and 0.366 (nd6).

When we calculated ω values for terminal branches to measure the strength of selection between different locomotive abilities (i.e., non-flying vs. flying insects), it was not found any significant differences in ω values between two groups of insects (Figure 1).

**Figure 2:**
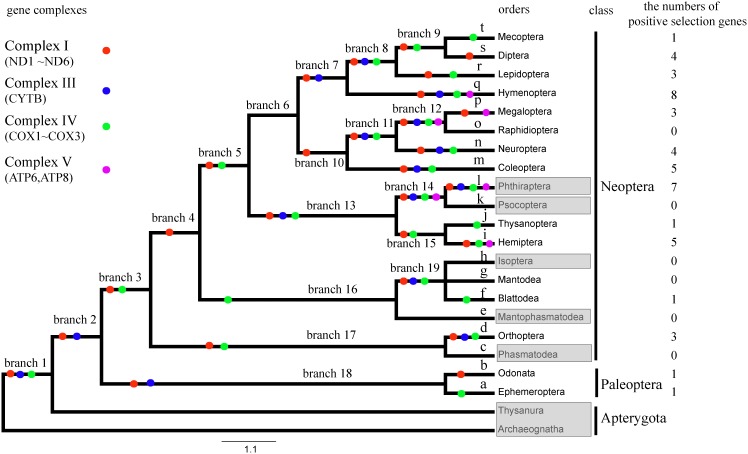
Positive selection detected by branch site model of mtDNA in each branch of insects. Nd1, nd2, nd3, nd4, nd4l, nd5, and nd6 belong to complex I (red circle), cyt b belongs to complex III (blue circle), cox1, cox2, and cox3 belong to complex IV (green), and atp6 and atp8 belong to complex V (pink). 12 of 13 mtDNAs were under positive selection, with the exception being nd3. Flightless insect orders are marked in gray. There is nearly no detectable positive selective pressure acting on flying insects. The numbers of genes in the terminal branch in each order (a–t) where positive selection was detected are shown in the last column. The number of positively selected genes is nearly three times higher in indirect flight insects (24.36%) than direct flight insects (8.33%). Branches 1–19 are the ancestor branches during insect evolution.

To test whether heterogeneous selective pressures act on specific branches, particularly on those branches with strong flight ability, we used the branch model implemented in PAML. First, we evaluated selective pressures acting on flying insects (foreground, ω_1_) and non-flying insects (background, ω_0_) using the two-ratio branch model. The LRT tests showed that the two-ratio model fits were significantly better than the one-ratio model at eight genes (i.e., atp6, p = 0.018; cox1, p = 0.001; nd1, p = 0.002; nd2, p = 0.007; nd4, p = 0.001; nd4l, p = 0.007; nd5, p<0.001, Table 1), indicating a divergence in selective pressure between flying and non-flying insects. We next evaluated selective pressure acting on the LCA of Pterygota that possessed primordial flight ability. The LRT tests comparing one ratio models and two ratio models showed that the two-ratio models were significantly better than the one-ratio models for six genes (cox1, p<0.001; cyt b, p = 0.012; nd1, p = 0.042; nd2, p = 0.009; nd4, p<0.001; nd5, p<0.001; Table 2, Table S2), suggesting that positive selection acted on mtDNA protein-encoding genes to accommodate the energy demands that accompanied the appearance of flight ability. Finally, when insect orders (a–t, Figure 2) were treated as a separate foreground branch, the results showed that insects with different flight abilities experienced different selective pressures acting on mtDNA protein-encoding genes. In particular, infinite ω (i.e., *d_S_* = 0 and *d_N_*>0) or ω>1, which may represent positive selection, were limited to eleven orders: Ephemeroptera (a: cox1), Phasmatodea (c: cox1), Mantophasmatodea (e: nd4), Blattodea (f: cox1), Mantodea (g: nd1, nd4, nd5), Isoptera (h: nd2, nd4l, cox1), Thysanoptera (j: cyt b, nd4, cox1), Neuroptera (n: cyt b, cox1), Raphidioptera (o: nd1, nd5, cox2), Lepidoptera (r: nd4, cox1), Mecoptera (t: nd3, nd4, nd5), eight of which have flight ability, e.g., Ephemeroptera, Blattodea, Mantodea, Thysanoptera, Neuroptera, Raphidioptera, Lepidoptera, Mecoptera.

**Table 2:** CodeML analysis of mitochondrial protein-encoding genes (13 genes) and evidence of positive selection of cox1, cyt b, nd4 and nd5 in flying insects.

Gene	Model*	-lnL	Model compare	2ΔlnL	df	P value	Parameters	Positive site (p>80%)
**cox1**	M0: oneratio	57994.153					k = 1.475 ω = 0.022	
	branch 3							
	A: tworatio	57984.358	A VS M0	19.591	1	0.000	k = 1.48134 ω_0_ = 0.021 ω_1_ = 126.330	
	B: tworatio fixω = 1	57984.601	A VS B	0.487	1	0.485	k = 1.48134 ω_0_ = 0.021 ω_1_ = 1.000	
	MA	57551.109					k = 1.646 p_0_ = 0.900 p_1_ = 0.049p_2a_ = 0.048 p_2b_ = 0.003 ω_0 = _0.021 ω_1_ = 1.000ω_2_ = 15.295	388 (0.837) 394 (0.866) 431 (0.875) 472 (0.999) 474 (0.812) 475 (0.988)
	MA0	57553.125	MA VS MA0	4.032	1	0.045	k = 1.644 p_0_ = 0.847 p_1_ = 0.046p_2a_ = 0.102 p_2b_ = 0.006 ω_0 = _0.021 ω_1_ = 1.000ω_2_ = 1.000	
**cyt b**	M0: oneratio	49988.410					k = 1.385 ω = 0.035	
	branch 2							
	A: tworatio	49985.264	A VS M0	6.292	1	0.012	k = 1.389 ω_0 = _0.034ω_1_ = 999.000	
	B: tworatio fixω = 1	499853409	A VS B	0.255	1	0.614	k = 1.389 ω_0 = _0.035ω_1_ = 1.000	
	MA	49602.136					k = 1.454 p_0_ = 0.932 p_1_ = 0.051p_2a_ = 0.015 p_2b_ = 0.001 ω_0 = _0.034 ω_1_ = 1.000ω_2_ = 999.000	2 (0.969)
	MA0	49606.827	MA VS MA0	9.382	1	0.002	k = 1.451 p_0_ = 0.842p_1_ = 0.046 p_2a_ = 0.105 p_2b_ = 0.006 ω_0 = _0.034 ω_1_ = 1.000 ω_2_ = 1.000	
**nd4**	M0: oneratio	69771.825					k = 1.005 ω = 0.033	
	branch 2							
	A: tworatio	69766.087	A VS M0	11.478	1	0.001	k = 1.005 ω_0 = _0.033 ω_1_ = 999.000	
	B: tworatio fix ω = 1	69766.294	A VS B	0.421	1	0.516	k = 1.005 ω_0 = _0.033 ω_1_ = 1.000	
	MA	69125.348					k = 0.972 p_0_ = 0.821 p_1_ = 0.078p_2a_ = 0.092 p_2b_ = 0.009 ω_0 = _0.035 ω_1_ = 1.000ω_2_ = 999.000	186 (0.907)
	MA0	69127.742	MA VS MA0	4.788	1	0.0286	k = 0.972 p_0_ = 0.815 p_1_ = 0.077p_2a_ = 0.099 p_2b_ = 0.009 ω_0 = _0.035 ω_1_ = 1.000 ω_2_ = 1.000	
	branch 3							
	A: two ratio	69767.585	A VS M0	8.481	1	0.004	k = 1.007 ω_0 = _0.033 ω_1_ = 520.490	
	B: two ratio fix ω = 1	69767.642	A VS B	0.114	1	0.735	k = 1.007 ω_0 = _0.033 ω_1_ = 1.000	
	MA	69121.836					k = 0.974 p_0_ = 0.877 p_1_ = 0.083p_2a_ = 0.036 p_2b_ = 0.003 ω_0 = _0.035 ω_1_ = 1.000 ω_2_ = 21.114	30 (0.961) 78 (0.962) 119 (0.997) 180 (0.986) 380 (0.995) 383 (0.929)
	MA0	69127.578	MA VS MA0	11.484	1	0.0007	k = 0.972 p_0_ = 0.851 p_1_ = 0.081p_2a_ = 0.062 p_2b_ = 0.006 ω_0 = _0.035 ω_1_ = 1.000 ω_2_ = 1.000	
**nd5**	M0: one ratio	90180.389					k = 1.048 ω = 0.027	
	branch 2							
	A: two ratio	90175.002	A VS M0	10.773	1	0.001	k = 1.048 ω_0 = _0.027 ω_1_ = 999.000	
	B: tworatio fix ω = 1	90175.212	A VS B	0.420	1	0.517	k = 1.048 ω_0 = _0.027 ω_1_ = 1.000	
	MA	89252.419					k = 1.312 p_0_ = 0.812 p_1_ = 0.188p_2a_ = 0.000 p_2b_ = 0.000 ω_0 = _0.050 ω_1_ = 1.000 ω_2_ = 1.000	155 (0.897) 253 (0.873)
	MA0	89249.384	MA VS MA0	6.072	1	0.0137	k = 1.313 p_0_ = 0.715 p_1_ = 0.165p_2a_ = 0.098 p_2b_ = 0.023 ω_0 = _0.049 ω_1_ = 1.000 ω_2_ = 1.000	

*Branch 2 and branch 3 represent the LCA of Pterygota and LCA of Neoptera, respectively.

Considering that positive selection sometimes acts only on a few sites and within a short evolutionary time period, branch-site models were used to identify positively selected sites in each evolutionary lineage (branch 1–19 and a–t in Figure 2). Although positive selection was detected for at least one gene of nearly all branches (Table S4), stronger selection was found in those branches of insects that have stronger flight ability. Particularly, the LCA of Pterygota (branch 2), which displayed initial flight ability in insects, showed that the alternative model (MA) fitted the data better than the null model (MA0) for cyt b, nd4 and nd5 (cyt b, p = 0.002; nd4, p = 0.029; nd5, p = 0013; Table 2) with ω>1, and four amino acid sites identified to be under positive selection (posterior probability≥80%) for cyt b (n = 1), nd4 (n = 1) and nd5 (n = 2). The LRT tests of the branch-site model on each order (a–t in Figure 2) also showed evidence of positive selection in most insect orders with strong flight ability after correcting for multiple testing, i.e., branch a, b, d, f, i, j, l–n, p–t (p<0.05, Figure 2 and Table S3), while the other two orders, Raphidioptera (m) and Mantodea (e), showed no sign of positive selection, although they also have strong flight ability. When only insects with degenerated wings, including Phthiraptera (l), Psocoptera (k the sampled species has no flight ability), Isoptera (h), Mantophasmatodea (e) and Phasmatodea (c) were considered, all except for Phthiraptera (l) showed no significant sign of positive selection (p>0.05, Figure 2 and Table S3).

### Selective Pressure on Insects with different Locomotive Patterns

Flying insects have two locomotive modes, indirect flight and direct flight. Treating indirect flight insects as the foreground and direct flight insects as the background, the two-ratio model had a significantly better fit than the one-ratio model that compared indirect flight and direct flight insects (p<0.05; Table S2), indicating that the two locomotive modes experienced different evolutionary pressures. Besides the different pressure between indirect flight and direct flight insects, the estimated ω values showed that only the ‘a’ branch, which belongs to the direct flight mode, underwent positive selection, whereas all other positively selected orders belong to the indirect flight mode.

Positive selection in indirect flight insect genes was further supported by the branch-site model (Table S3). Six positively selected sites were detected in the LCA of Neoptera (branch 3) at both cox1 (p = 0.045) and nd4 (p<0.001) (Table 2). In addition, the number of mtDNA protein-encoding genes identified to be under positive selection in Palaeoptera with direct flight (8.33%) was much lower than that in Neoptera with indirect flight (24.36%) (Figure 2 and Table S3), suggesting a stronger selective pressure for the indirect flight pattern.

## Discussion

### Stronger Adaptive Evolution in Flying Insects

Along with the evolution of flight ability, winged insects (Pterygota) became the most successful group of terrestrial arthropods [23], [29]. Flight is an energetically costly activity as shown by physiologic studies that found that in insects the flight metabolic rate is higher than the rate at rest [30]. For example, *Apis mellifera* of order Hymenoptera has a much higher energy expenditure while flying (87.72 mm^3^O_2_/mg/h of FMR) than resting (3.21 mm^3^O_2_/mg/h RMR) [31], [32]. Although advantages associated with flight, such as the ability to disperse widely and forage are beneficial to survival, flight ability has been independently lost in some orders of Pterygota [33]. During the appearance or loss of wings and associated flight ability, a dramatic change in energy consumption, either increasing or decreasing, was suggested to occur [34], [35].

Mitterboeck and Adamowicz [16] found relaxed selective constraints at mtDNA protein-coding loci with a significant pattern of higher *dN/dS* ratios in flightless lineages. However, this was not corroborated in the present study because it was not found find statistically higher ω values in non-flying insects than in flying insects for all genes examined here (Figure 1). Different with Mitterboeck and Adamowicz’s study [16], the present study focused on both flying and non-flying insects and found some strong signals of positive selection and adaptive evolution in the flying insects, which could provide some novel insights into understanding the evolution mechanism of flight ability in insects.

This study revealed significant positive selection in mitochondrial protein-encoding genes of flying insects. For example, cyt b, nd4 and nd5 were subjected to strong positive selection leading to the LCA of Pterygota, which evolved the initial flight ability of insects (Table 2). Notably, more significant positive selection was identified in insects with stronger flight ability, suggesting that adaptive evolution of mtDNA protein-encoding genes might occur in response to increased energy consumption in flying insects. A similar pattern was also found in ancestors of flying bats, which showed evidence of adaptive evolution of nd4, cyt b, and atp8 [6].

Significant positive selection was unexpectedly observed in Phthiraptera, an order having no flight ability. Johnson et al.[36] found that rate of mitochondrial substitution is elevated in lice. Significant non-synonymous substitution rates may arise through oxidative stress or the DNA repair machinery. Phthiraptera are ectoparasites of birds and mammals that feed on blood [37]. Considering the effect of blood meals on functional and structural changes in mitochondria of *Aedes aegypti* revealed in a previous study [38], positive selection with significant non-synonymous substitution rates in this order may thus represent an important adaptation to their special feeding habit. However the further analyses and additional insight into the possible molecular adaptation of mitochondrial DNA in response to blood feeding habits in insects is needed.

### Enhanced Adaptive Evolution in Indirect Flight Insects

The evolutionarily successful insect groups such as Coleoptera, Diptera and Hymenoptera may be attributed in part to the evolution of asynchronous flight muscle [39]. The indirect flight locomotive mode expands on the direct flight mode through the presence of muscles that allow insects to fly backward and hover. The wing-beat frequencies ranged from 5 to 200 Hz in insects with synchronous direct flight muscles, whereas those of indirect flight insects with an asynchronous flight mechanism may exceed 1000 Hz [40], [41]. Such high-frequency operation indicates that asynchronous muscles likely have a higher mass-specific energy output than do synchronous muscles [42], [43].

Previous studies showed that mammalian species with specialized locomotive modes, including diving cetaceans, flying bats, and alpacas at high altitude, underwent adaptive evolution [5]–[7]. A similar pattern was also found in insects. The present study provided evidence to support that the change in locomotive pattern from direct flight to indirect flight is associated with positive selection at cox1 and nd4. Also, the selective pressure for indirect flight insects was higher than for direct flight insects as evidenced by the proportion of mtDNA protein-encoding genes under positive selection (24.36% in indirect flight insects, Neoptera vs. 8.33% in direct flight insects, Pterygota). The Neoptera insects are more flexible and need more energy than Pterygota during flying. The more positive selection detected in Neoptera than in Pterygota is in accordance with behavioral and physiological evidence showing that flight activity intensity as expressed by wing-beat frequency in indirect flight insects was higher than that for direct flight insects.

Besides the LCAs of Pterygota and Neoptera, the positive selection occurs in nearly whole evolution process of insects and in nearly each mitochondrial protein encoding genes. The ancestor of insects has no wings [10], and along with the evolution of insects, the appearance of wings is the most significant feature. The development of wings offered insects the opportunity to occupy wider ecological niches. In the process of wings’ development, the mitochondrial protein encoding genes may have undergone continued evolution (positive selection) in order to increase their energy supply to improve the flight ability. The variation in the selective pressure on OXPHOS complexes may be related with their difference in functional significance. For example, complex I acts as the first step to initiate the OXPHOS, whereas complex IV seems to be more important in energy supply compared with other complexes. Physiological studies have detected each complex in OXPHOS, and found that the free energy supply of complex IV (cytochrome oxidase, 100 kJ/mole) is twice as high compared to complex I (NADH dehydrogenase, 52 kJ/mole) and complex III (cytochrome bc 1, 42 kJ/mole) [44]. It was also suggested a regulatory role of complex IV in the electron transport chain of mitochondria or OXPHOS. These can in part explain why we detected relatively more selection in complexes I and IV, but more evidences, especially some functional experiments, are needed to validate these conjectures.

In conclusion, this study revealed several indications of positive selection of mitochondrial protein-encoding genes belonging to the OXPHOS pathway. The pattern of positive selection is closely associated with the appearance of flight ability in the early evolutionary stage of insects and changes in flight modes from direct to indirect. This study could provide insight into the molecular mechanism of flight evolution in insects, and thus increase the understanding of their exceptional adaptation to various habitats.

## Supporting Information

Figure S1 **A well-supported phylogenetic tree used for selective pressure in PAML analysis.** The phylogenetic relationship among major insect groups is based on previous studies. And different orders of insects are marked with different colors.
(TIF)

Table S1 Sequence data used in this study, including taxonomy and accession numbers.
(DOC)

Table S2 CodeML analyses of selective patterns for mtDNA genes in insects with branch and branch site models on LCA of Pterygota and LCA of Neoptera.
(DOC)

Table S3 Evidence of positive selection for mtDNA genes in insect orders with branch site model.
(DOC)

Table S4 Evidence of positive selection for mtDNA genes of each ancestor branch with branch site model.
(DOC)
