# Supplementary figures and images for: Adaptive Evolution of Mitochondrial Energy Metabolism Genes Associated with Increased Energy Demand in Flying Insects
Source: PLoS One. 2014 Jun 11;9(6):e99120. doi: 10.1371/journal.pone.0099120 (PMC4053383; doi:10.1371/journal.pone.0099120)

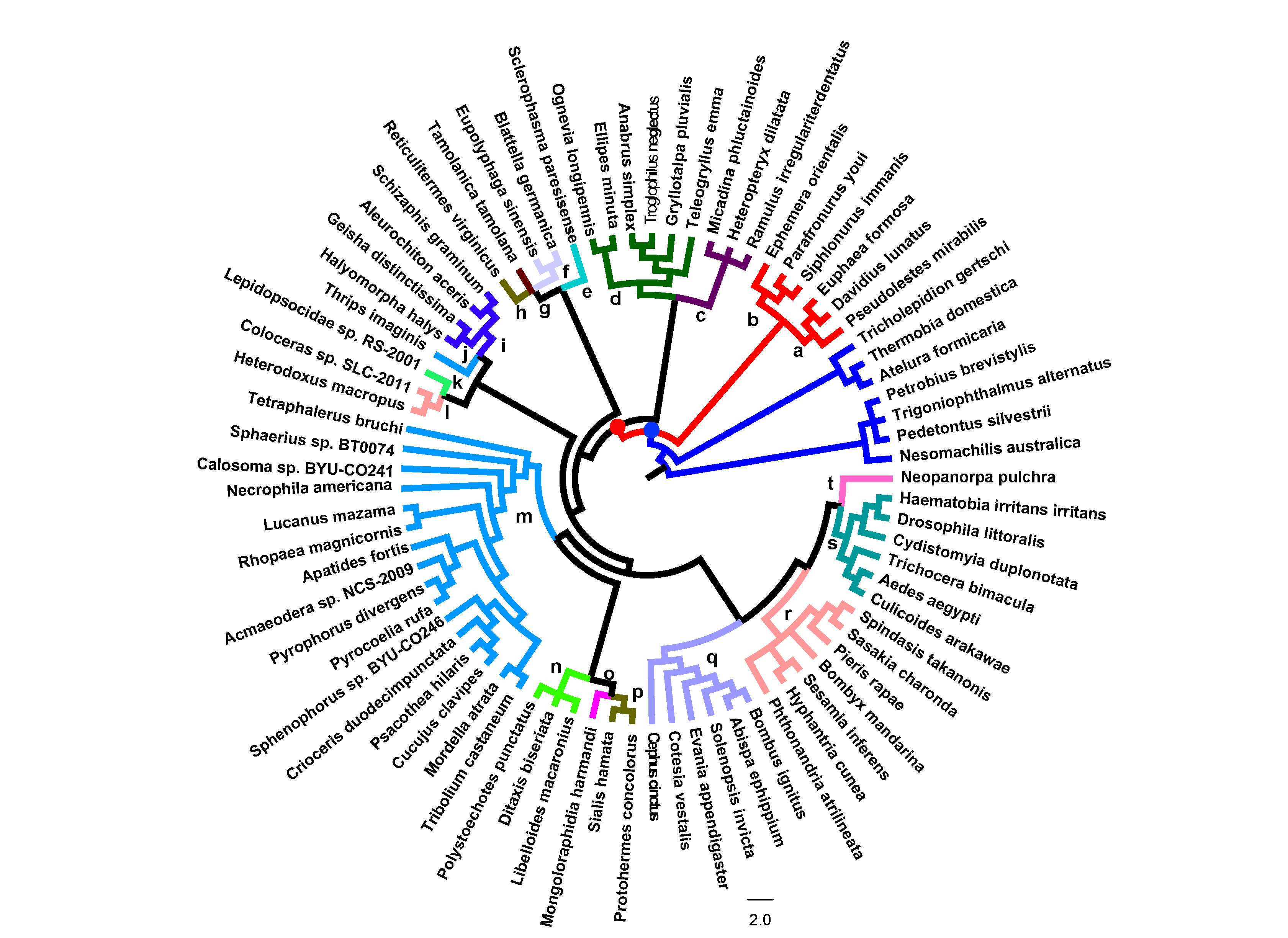

Supplement: Figure S1 — A well-supported phylogenetic tree used for selective pressure in PAML analysis. The phylogenetic relationship among major insect groups is based on previous studies. And different orders of insects are marked with different colors. (TIF) [file pone.0099120.s001.tif]
