# Supplementary material for: Adaptive Evolution of Mitochondrial Energy Metabolism Genes Associated with Increased Energy Demand in Flying Insects
Source: PLoS One. 2014 Jun 11;9(6):e99120. doi: 10.1371/journal.pone.0099120 (PMC4053383; doi:10.1371/journal.pone.0099120)
Supplement: Table S1 — Sequence data used in this study, including taxonomy and accession numbers. (DOC) [file pone.0099120.s002.doc]

**Table** **S1 Sequence data used in this study, including taxonomy and accession numbers**

| **Subclass** | **Order** | **family** | **genus** | **Species_name** | **Access number** | **Sign*** |
| --- | --- | --- | --- | --- | --- | --- |
| Apterygota |  |  |  |  |  |  |
|  | Archaeognatha |  |  |  |  |  |
|  |  | Meinertellidae | Nesomachilis | *Nesomachilis australica* | NC_006895 | 0 |
|  |  | Machilidae | Trigoniophthalmus | *Trigoniophthalmus alternatus* | EU016193 | 0 |
|  |  | Machilidae | Pedetontus | *Pedetontus silvestrii* | EU621793 | 0 |
|  |  | Machilidae | Petrobius | *Petrobius brevistylis* | NC_007688 | 0 |
|  | Thysanura |  |  |  |  |  |
|  |  | Lepidotrichidae | Tricholepidion | *Tricholepidion gertschi* | NC_005437 | 0 |
|  |  | Nicoletiidae | Atelura | *Atelura formicaria* | EU084035 | 0 |
|  |  | Lepismatidae | Thermobia | *Thermobia domestica* | NC_006080 | 0 |
| Palaeoptera |  |  |  |  |  |  |
|  | Ephemeroptera(a) |  |  |  |  |  |
|  |  | Heptageniidae | Parafronurus | *Parafronurus youi* | EU349015 | 1 |
|  |  | Siphlonuridae | Siphlonurus | *Siphlonurus immanis* | FJ606783 | 1 |
|  |  | Ephemeridae | Ephemera | *Ephemera orientalis* | EU591678 | 1 |
|  | Odonata(b) |  |  |  |  |  |
|  |  | Pseudolestidae | Pseudolestes | *Pseudolestes mirabilis* | FJ606784 | 1 |
|  |  | Gomphidae | Davidius | *Davidius lunatus* | EU591677 | 1 |
|  |  | Euphaeidae | Euphaea | *Euphaea formosa* | HM126547 | 1 |
| Neoptera |  |  |  |  |  |  |
|  | Phasmatodea (c) |  |  |  |  |  |
|  |  | Diapheromeridae | Micadina | *Micadina phluctainoides* | AB477466 | 0 |
|  |  | Heteropterygidae | Heteropteryx | *Heteropteryx dilatata* | AB477468 | 0 |
|  |  | Phasmatidae | Ramulus | *Ramulus irregulariterdentatus* | AB477463 | 0 |
|  | Orthoptera (d) |  |  |  |  |  |
|  |  | Acrididae | Ognevia | *Ognevia longipennis* | EU914848 | 1 |
|  |  | Gryllidae | Teleogryllus | *Teleogryllus emma* | EU557269 | 1 |
|  |  | Gryllotalpidae | Gryllotalpa | *Gryllotalpa pluvialis* | EU938371 | 0 |
|  |  | Rhaphidophoridae | Troglophilus | *Troglophilus neglectus* | EU938374 | 0 |
|  |  | Tettigoniidae | Anabrus | *Anabrus simplex* | NC_009967 | 0 |
|  |  | Tridactylidae | Ellipes | *Ellipes minuta* | GU945502 | 0 |
|  | Isoptera (h) |  |  |  |  |  |
|  |  | Rhinotermitidae | Reticulitermes | *Reticulitermes virginicus* | NC_009500 | 0 |
|  | Mantodea (g) |  |  |  |  |  |
|  |  | Mantidae | Tamolanica | *Tamolanica tamolana* | NC_007702 | 1 |
|  | Mantophasmatodea (e) |  |  |  |  |  |
|  |  | Mantophasmatidae | Sclerophasma | *Sclerophasma paresisense* | NC_007701 | 0 |
|  | Blattodea (f) |  |  |  |  |  |
|  |  | Ectobiidae | Blattella | *Blattella germanica* | EU854321 | 0 |
|  |  | Corydiidae | Eupolyphaga | *Eupolyphaga sinensis* | FJ830540 | 0 |
|  | Phthiraptera (l) |  |  |  |  |  |
|  |  | Boopidae | Heterodoxus | *Heterodoxus macropus* | NC_002651 | 0 |
|  |  | Philopteridae | Coloceras | Coloceras sp. SLC-2011 | JN122000 | 0 |
|  | Psocoptera (k) |  |  |  |  |  |
|  |  | Lepidopsocidae | -- | Lepidopsocidae sp. RS-2001 | NC_004816 | 0 |
|  | Thysanoptera (j) |  |  |  |  |  |
|  |  | Thripidae | -- | *Thrips imaginis* | NC_004371 | 1 |
|  | Hemiptera (i) |  |  |  |  |  |
|  |  | Aleyrodidae | Aleurochiton | *Aleurochiton aceris* | NC_006160 | 1 |
|  |  | Aphididae | Schizaphis | *Schizaphis graminum* | NC_006158 | 0 |
|  |  | Flatidae | Geisha | *Geisha distinctissima* | FJ230961 | 1 |
|  |  | Pentatomidae | Halyomorpha | *Halyomorpha halys* | FJ685650 | 1 |
|  | Raphidioptera (o) |  |  |  |  |  |
|  |  | Raphidiidae | Mongoloraphidia | *Mongoloraphidia harmandi* | FJ859902 | 1 |
|  | Megaloptera (p) |  |  |  |  |  |
|  |  | Corydalidae | Protohermes | *Protohermes concolorus* | EU526394 | 1 |
|  |  | Sialidae | Sialis | *Sialis hamata* | FJ859905 | 1 |
|  | Neuroptera (n) |  |  |  |  |  |
|  |  | Ascalaphidae | Libelloides | *Libelloides macaronius* | FR669150 | 1 |
|  |  | Mantispidae | Ditaxis | *Ditaxis biseriata* | FJ859906 | 1 |
|  |  | Polystoechotidae | Polystoechotes | *Polystoechotes punctatus* | FJ171325 | 1 |
|  | Coleoptera (m) |  |  |  |  |  |
|  |  | Bostrichidae | Apatides | *Apatides fortis* | FJ613421 | 0 |
|  |  | Buprestidae | Acmaeodera | Acmaeodera sp. NCS-2009 | FJ613420 | 1 |
|  |  | Carabidae | Calosoma | Calosoma sp. BYU-CO241 | GU176340 | 0 |
|  |  | Cerambycidae | Psacothea | *Psacothea hilaris* | FJ424074 | 1 |
|  |  | Chrysomelidae | Crioceris | *Crioceris duodecimpunctata* | NC_003372 | 1 |
|  |  | Cucujidae | Cucujus | *Cucujus clavipes* | GU176341 | 0 |
|  |  | Curculionidae | Sphenophorus | Sphenophorus sp. BYU-CO246 | GU176342 | 0 |
|  |  | Elateridae | Pyrophorus | *Pyrophorus divergens* | NC_009964 | 1 |
|  |  | Lampyridae | Pyrocoelia | *Pyrocoelia rufa* | NC_003970 | 1 |
|  |  | Lucanidae | Lucanus | *Lucanus mazama* | FJ613419 | 0 |
|  |  | Mordellidae | Mordella | *Mordella atrata* | FJ859904 | 1 |
|  |  | Ommatidae | Tetraphalerus | *Tetraphalerus bruchi* | EU877953 | 1 |
|  |  | Scarabaeidae | Rhopaea | *Rhopaea magnicornis* | FJ859903 | 1 |
|  |  | Silphidae | Necrophila | *Necrophila americana* | GU176343 | 0 |
|  |  | Sphaeriusidae | Sphaerius | *Sphaerius sp. BT0074* | EU877950 | 0 |
|  |  | Tenebrionidae | Tribolium | *Tribolium castaneum* | NC_003081 | 0 |
|  | Lepidoptera (r) |  |  |  |  |  |
|  |  | Arctiidae | Hyphantria | *Hyphantria cunea* | GU592049 | 1 |
|  |  | Bombycidae | Bombyx | *Bombyx mandarina* | NC_003395 | 0 |
|  |  | Geometridae | Phthonandria | *Phthonandria atrilineata* | EU569764 | 1 |
|  |  | Lycaenidae | Spindasis | *Spindasis takanonis* | HQ184266 | 1 |
|  |  | Noctuidae | Sesamia | *Sesamia inferens* | JN039362 | 1 |
|  |  | Nymphalidae | Sasakia | *Sasakia charonda* | AP011824 | 1 |
|  |  | Pieridae | Pieris | *Pieris rapae* | HM156697 | 1 |
|  | Mecoptera (t) |  |  |  |  |  |
|  |  | Panorpidae | Neopanorpa | *Neopanorpa pulchra* | FJ169955 | 1 |
|  | Diptera (s) |  |  |  |  |  |
|  |  | Ceratopogonidae | Culicoides | *Culicoides arakawae* | NC_009809 | 1 |
|  |  | Culicidae | Aedes | *Aedes aegypti* | NC_010241 | 1 |
|  |  | Drosophilidae | Drosophila | *Drosophila littoralis* | FJ447340 | 1 |
|  |  | Muscidae | Haematobia | *Haematobia irritans irritans* | NC_007102 | 1 |
|  |  | Tabanidae | Cydistomyia | *Cydistomyia duplonotata* | NC_008756 | 1 |
|  |  | Trichoceridae | Trichocera | *Trichocera bimacula* | JN861750 | 1 |
|  | Hymenoptera (q) |  |  |  |  |  |
|  |  | Apidae | Bombus | *Bombus ignitus* | DQ870926 | 1 |
|  |  | Braconidae | Cotesia | *Cotesia vestalis* | FJ154897 | 1 |
|  |  | Cephidae | Cephus | *Cephus cinctus* | FJ478173 | 1 |
|  |  | Evaniidae | Evania | *Evania appendigaster* | FJ593187 | 1 |
|  |  | Formicidae | Solenopsis | *Solenopsis invicta* | HQ215538 | 0 |
|  |  | Vespidae | Abispa | *Abispa ephippium* | EU302588 | 1 |

***:** 0 and 1 represent non flying and flying, respectively.
