# Supplementary material for: Adaptive Evolution of Mitochondrial Energy Metabolism Genes Associated with Increased Energy Demand in Flying Insects
Source: PLoS One. 2014 Jun 11;9(6):e99120. doi: 10.1371/journal.pone.0099120 (PMC4053383; doi:10.1371/journal.pone.0099120)
Supplement: Table S2 — CodeML analyses of selective patterns for mtDNA genes in insects with branch and branch site models on LCA of Pterygota and LCA of Neoptera. (DOC) [file pone.0099120.s003.doc]

**Table S2 CodeML analyses of selective pattern for mtDNA genes in insects with branch and branch site models on LCA of Pterygota and LCA of Neoptera**

| gene | model* | -ln L | model compare | 2Δln | df | p | parameters | Positive site (p>80%) |
| --- | --- | --- | --- | --- | --- | --- | --- | --- |
| atp6 | M0: one ratio | 31983.802 |  |  |  |  | k = 0.962 ω = 0.026 |  |
|  | branch 2 |  |  |  |  |  |  |  |
|  | A: two ratio | 31982.323 | A VS M0 | 2.958 | 1 | 0.085 | k = 0.961 ω_0_ = 0.026 ω_1_ = 999.000 |  |
|  | B: two ratio fix ω=1 | 31982.376 | A VS B | 0.107 | 1 | 0.744 | k = 0.961 ω_0_ = 0.026 ω_1_ = 1.000 |  |
|  | MA | 31542.394 |  |  |  |  | k = 0.990 p_0_ =0.003 p_1_ = 0.000 p_2a_ = 0.907 p_2b_ = 0.090ω_0 =_ 0.033 ω_1_ = 1.000 ω_2_ = 14.24389 |  |
|  | MA0 | 31542.406 | MA VS MA0 | 0.024 | 1 | 0.887 | k = 0.990 p_0_ = 0.001 p_1_ = 0.000 p_2a_ = 0.909 p_2b_ = 0.090ω_0 =_ 0.033 ω_1_ = 1.000 ω_2_ = 1.000 |  |
|  | branch 3 |  |  |  |  |  |  |  |
|  | A: two ratio | 31981.275 | A VS M0 | 5.054 | 1 | 0.024 | k = 0.963 ω_0_ = 0.025 ω_1_ = 999.000 |  |
|  | B: two ratio fix ω=1 | 31981.341 | A VS B | 0.131 | 1 | 0.717 | k = 0.962 ω_0_ = 0.025 ω_1_ = 1.000 |  |
|  | MA | 31543.284 |  |  |  |  | k = 0.990 p_0_ = 0.890 p_1_ = 0.088 p_2a_ = 0.019 p_2b_ = 0.002ω_0 =_ 0.033 ω_1_ = 1.000 ω_2_ = 1.000 |  |
|  | MA0 | 31543.284 | MA VS MA0 | 0 | 1 | 1 | k = 0.990 p_0_ = 0.890 p_1_ = 0.088 p_2a_ = 0.019 p_2b_ = 0.002ω_0 =_ 0.033 ω_1_ = 1.000 ω_2_ = 1.000 |  |
| atp8 | M0: one ratio | 9970.289 |  |  |  |  | k = 1.476 ω = 0.009 |  |
|  | branch 2 |  |  |  |  |  |  |  |
|  | A: two ratio | 9970.285 | A VS M0 | 0.008 | 1 | 0.928 | k = 1.475 ω_0_ = 0.009 ω_1_ = 4.228 |  |
|  | B: two ratio fix ω=1 | 9970.286 | A VS B | 0.002 | 1 | 0.964 | k = 1.476 ω_0_ = 0.009 ω_1_ = 1.000 |  |
|  | MA | 9701.584 |  |  |  |  | k = 1.427 p_0_ = 0.422 p_1_ = 0.510 p_2a_ = 0.031 p_2b_ = 0.037ω_0 =_ 0.085 ω_1_ = 1.000 ω_2_ = 688.310 | 20 (0.982) |
|  | MA0 | 9702.752 | MA VS MA0 | 2.336 | 1 | 0.127 | k = 1.423 p_0_ = 0.396 p_1_ = 0.478 p_2a_ = 0.057 p_2b_ = 0.069ω_0 =_ 0.085 ω_1_ = 1.000 ω_2_ = 1.000 |  |
|  | branch 3 |  |  |  |  |  |  |  |
|  | A: two ratio | 9970.289 | A VS M0 | 0.000 | 1 | 1.000 | k = 1.476 ω_0_ = 0.009 ω_1_ = 0.610 |  |
|  | B: two ratio fix ω=1 | 9970.289 | A VS B | 0.000 | 1 | 1.000 | k = 1.476 ω_0_ = 0.009 ω_1_ = 1.000 |  |
|  | MA | 9703.925 |  |  |  |  | k = 1.435 p_0_ = 0.453 p_1_ = 0.547 p_2a_ = 0.000 p_2b_ = 0.000ω_0 =_ 0.085 ω_1_ = 1.000 ω_2_ = 1.000 |  |
|  | MA0 | 9703.925 | MA VS MA0 | 0 | 1 | 1 | k = 1.435 p_0_ = 0.369 p_1_ = 0.447 p_2a_ = 0.083 p_2b_ = 0.101ω_0 =_ 0.085 ω_1_ = 1.000 ω_2_ = 1.000 |  |
| cox1 | M0: one ratio | 57994.153 |  |  |  |  | k = 1.475 ω = 0.022 |  |
|  | branch 2 |  |  |  |  |  |  |  |
|  | A: two ratio | 57984.001 | A VS M0 | 20.304 | 1 | 0.000 | k = 1.478 ω_0_ = 0.021 ω_1_ = 999.000 |  |
|  | B: two ratio fix ω=1 | 57984.212 | A VS B | 0.421 | 1 | 0.516 | k = 1.478 ω_0_ = 0.021 ω_1_ = 1.000 |  |
|  | MA | 57552.514 |  |  |  |  | k = 1.643 p_0_ = 0.879 p_1_ = 0.048 p_2a_ = 0.070 p_2b_ = 0.004ω_0 =_ 0.021 ω_1_ = 1.000 ω_2_ = 999.000 | 43 (0.993) 112 (0.852) 478 (0.899) |
|  | MA0 | 57554.252 | MA VS MA0 | 3.476 | 1 | 0.0622 | k = 1.642p_0_ = 0.842 p_1_ = 0.046 p_2a_ = 0.107 p_2b_ = 0.006ω_0 =_ 0.021 ω_1_ = 1.000 ω_2_ = 1.000 |  |
|  | branch 3 |  |  |  |  |  |  |  |
|  | A: two ratio | 57984.358 | A VS M0 | 19.591 | 1 | 0.000 | k = 1.48134 ω_0_ = 0.021 ω_1_ = 126.330 |  |
|  | B: two ratio fix ω=1 | 57984.601 | A VS B | 0.487 | 1 | 0.485 | k = 1.48134 ω_0_ = 0.021 ω_1_ = 1.000 |  |
|  | MA | 57551.109 |  |  |  |  | k = 1.646p_0_ = 0.900 p_1_ = 0.049 p_2a_ = 0.048 p_2b_ = 0.003ω_0 =_ 0.021 ω_1_ = 1.000 ω_2_ = 15.295 | 388 (0.837) 394 (0.866) 431 (0.875) 472 (0.999) 474 (0.812) 475 (0.988) |
|  | MA0 | 57553.125 | MA VS MA0 | 4.032 | 1 | 0.0446 | k = 1.644 p_0_ = 0.847 p_1_ = 0.046 p_2a_ = 0.102 p_2b_ = 0.006 ω_0 =_ 0.021 ω_1_ = 1.000 ω_2_ = 1.000 |  |
| cox2 | M0: one ratio | 30070.700 |  |  |  |  | k = 1.54 ω = 0.026 |  |
|  | branch 2 |  |  |  |  |  |  |  |
|  | A: two ratio | 30069.128 | A VS M0 | 3.145 | 1 | 0.076 | k = 1.536 ω_0_ = 0.026 ω_1_ = 999.000 |  |
|  | B: two ratio fix ω=1 | 30069.183 | A VS B | 0.110 | 1 | 0.740 | k = 1.536 ω_0_ = 0.026 ω_1_ = 1.000 |  |
|  | MA | 29853.981 |  |  |  |  | k = 1.639 p_0_ = 0.911 p_1_ = 0.038 p_2a_ = 0.049 p_2b_ = 0.002 ω_0 =_ 0.027 ω_1_ = 1.000 ω_2_ = 801.969 |  |
|  | MA0 | 29854.626 | MA VS MA0 | 0.645 | 1 | 0.422 | k = 1.639 p_0_ = 0.624 p_1_ = 0.026 p_2a_ = 0.336 p_2b_ = 0.014 ω_0 =_ 0.027 ω_1_ = 1.000 ω_2_ = 1.000 |  |
|  | branch 3 |  |  |  |  |  |  |  |
|  | A: two ratio | 30067.378 | A VS M0 | 6.644 | 1 | 0.009 | k = 1.534 ω_0_ = 0.026 ω_1_ = 999.000 |  |
|  | B: two ratio fix ω=1 | 30067.506 | A VS B | 0.254 | 1 | 0.614 | k = 1.534 ω_0_ = 0.026 ω_1_ = 1.000 |  |
|  | MA | 29849.231 |  |  |  |  | k = 1.637 p_0_ = 0.858 p_1_ = 0.036 p_2a_ = 0.102 p_2b_ = 0.004 ω_0 =_ 0.027 ω_1_ = 1.000 ω_2_ = 999.000 |  |
|  | MA0 | 29850.574 | MA VS MA0 | 2.686 | 1 | 0.1012 | k = 1.638 p_0_ = 0.768 p_1_ = 0.032 p_2a_ = 0.192 p_2b_ = 0.008 ω_0 =_ 0.027 ω_1_ = 1.000 ω_2_ = 1.000 |  |
| cox3 | M0: one ratio | 35894.893 |  |  |  |  | k = 1.467 ω = 0.03970 |  |
|  | branch 2 |  |  |  |  |  |  |  |
|  | A: two ratio | 35893.117 | A VS M0 | 3.552 | 1 | 0.059 | k = 1.470 ω_0_ = 0.039 ω_1_ = 4.334 |  |
|  | B: two ratio fix ω=1 | 35893.122 | A VS B | 0.009 | 1 | 0.924 | k = 1.470 ω_0_ = 0.039 ω_1_ = 1.000 |  |
|  | MA | 35617.500 |  |  |  |  | k = 1.678 p_0_ = 0.890 p_1_ = 0.092 p_2a_ = 0.017 p_2b_ = 0.002 ω_0 =_ 0.042 ω_1_ = 1.000 ω_2_ = 2.026 | 23 (0.884) |
|  | MA0 | 35617.599 | MA VS MA0 | 0.198 | 1 | 0.656 | k = 1.678 p_0_ = 0.884 p_1_ = 0.091 p_2a_ = 0.023 p_2b_ = 0.002 ω_0 =_ 0.042 ω_1_ = 1.000 ω_2_ = 1.000 |  |
|  | branch 3 |  |  |  |  |  |  |  |
|  | A: two ratio | 35891.769 | A VS M0 | 6.248 | 1 | 0.012 | k = 1.470 ω_0_ = 0.039 ω_1_ = 999.000 |  |
|  | B: two ratio fix ω=1 | 35891.888 | A VS B | 0.237 | 1 | 0.626 | k = 1.470 ω_0_ = 0.039 ω_1_ = 1.000 |  |
|  | MA | 35618.052 |  |  |  |  | k = 1.678 p_0_ = 0.905 p_1_ = 0.093 p_2a_ = 0.002 p_2b_ = 0.000 ω_0 =_ 0.042 ω_1_ = 1.000 ω_2_ = 1.000 |  |
|  | MA0 | 35618.052 | MA VS MA0 | 0 | 1 | 1 | k = 1.678 p_0_ = 0.907 p_1_ = 0.093 p_2a_ = 0.000 p_2b_ = 0.000 ω_0 =_ 0.042 ω_1_ = 1.000 ω_2_ = 1.000 |  |
| cytb | M0: one ratio | 49988.410 |  |  |  |  | k = 1.385 ω=0.035 |  |
|  | branch 2 |  |  |  |  |  |  |  |
|  | A: two ratio | 49985.264 | A VS M0 | 6.292 | 1 | 0.012 | k = 1.389 ω_0 =_ 0.034 ω_1_ = 999.000 |  |
|  | B: two ratio fix ω=1 | 499853409 | A VS B | 0.255 | 1 | 0.614 | k = 1.389 ω_0 =_ 0.035 ω_1_ = 1.000 |  |
|  | MA | 49602.136 |  |  |  |  | k = 1.454 p_0_ = 0.932 p_1_ = 0.051 p_2a_ = 0.015 p_2b_ = 0.001 ω_0 =_ 0.034 ω_1_ = 1.000 ω_2_ = 999.000 | 2 (0.969) |
|  | MA0 | 49606.827 | MA VS MA0 | 9.382 | 1 | 0.002 | k = 1.451 p_0_ = 0.842 p_1_ = 0.046 p_2a_ = 0.105 p_2b_ = 0.006 ω_0 =_ 0.034 ω_1_ = 1.000 ω_2_ = 1.000 |  |
|  | branch 3 |  |  |  |  |  |  |  |
|  | A: two ratio | 49987.383 | A VS M0 | 2.056 | 1 | 0.151 | k = 1.389 ω_0 =_ 0.035 ω_1_ = 0.146 |  |
|  | B: two ratio fix ω=1 | 49987.822 | A VS B | 0.879 | 1 | 0.348 | k = 1.389 ω_0 =_ 0.035 ω_1_ = 1.000 |  |
|  | MA | 49608.374 |  |  |  |  | k = 1.450 p_0_ = 0.930 p_1_ = 0.050 p_2a_ = 0.036 p_2b_ = 0.002 ω_0 =_ 0.034 ω_1_ = 1.000 ω_2_ = 3.501 |  |
|  | MA0 | 49608.787 | MA VS MA0 | 0.826 | 1 | 0.363 | k = 1.450 p_0_ = 0.911 p_1_ = 0.046 p_2a_ = 0.105 p_2b_ = 0.006 ω_0 =_ 0.034 ω_1_ = 1.000 ω_2_ = 1.000 |  |
| nd1 | M0: one ratio | 44931.572 |  |  |  |  | k = 1.080 ω = 0.030 |  |
|  | branch 2 |  |  |  |  |  |  |  |
|  | A: two ratio | 44929.495 | A VS M0 | 4.154 | 1 | 0.042 | k = 1.080 ω_1_ = 0.030 ω_2_ = 999.000 |  |
|  | B: two ratio fix ω=1 | 44929.575 | A VS B | 0.160 | 1 | 0.689 | k = 1.080 ω_1_ = 0.030 ω_2_ = 1.000 |  |
|  | MA | 44488.037 |  |  |  |  | k =1.221 p_0_ = 0.900 p_1_ = 0.094 p_2a_ = 0.005 p_2b_ = 0.001 ω_0 =_ 0.030 ω_1_ = 1.000 ω_2_ = 17.182 |  |
|  | MA0 | 44488.476 | MA VS MA0 | 0.878 | 1 | 0.349 | k = 1.222 p_0_ = 0.875 p_1_ = 0.092 p_2a_ = 0.030 p_2b_ = 0.003 ω_0 =_ 0.030 ω_1_ = 1.000 ω_2_ = 1.000 |  |
|  | branch 3 |  |  |  |  |  |  |  |
|  | A: two ratio | 44928.485 | A VS M0 | 6.176 | 1 | 0.013 | k = 1.080 ω_1_ = 0.030 ω_2_ = 999.000 |  |
|  | B: two ratio fix ω=1 | 44928.592 | A VS B | 0.200 | 1 | 0.655 |  |  |
|  | MA | 44487.529 |  |  |  |  | k = 1.220 p_0_ = 0.889 p_1_ = 0.093 p_2a_ = 0.016 p_2b_ = 0.002 ω_0 =_ 0.030 ω_1_ = 1.000 ω_2_ = 10.515 |  |
|  | MA0 | 44488.118 | MA VS MA0 | 1.178 | 1 | 0.278 | k = 1.221 p_0_ = 0.846 p_1_ = 0.089 p_2a_ = 0.059 p_2b_ = 0.006 ω_0 =_ 0.030 ω_1_ = 1.000 ω_2_ = 1.000 |  |
| nd2 | M0: one ratio | 63315.139 |  |  |  |  | k = 1.072 ω = 0.040 |  |
|  | branch 2 |  |  |  |  |  |  |  |
|  | A: two ratio | 63311.698 | A VS M0 | 6.883 | 1 | 0.009 | k = 1.071 ω_0 =_ 0.040 ω_1_ = 999.000 |  |
|  | B: two ratio fix ω=1 | 63311.856 | A VS B | 0.316 | 1 | 0.574 | k = 1.071 ω_0 =_ 0.040 ω_1_ = 1.000 |  |
|  | MA | 62774.922 |  |  |  |  | k = 1.150 p_0_ = 0.730 p_1_ = 0.127 p_2a_ = 0.121 p_2b_ = 0.021 ω_0 =_ 0.043 ω_1_ = 1.000 ω_2_ = 999.000 |  |
|  | MA0 | 62775.647 | MA VS MA0 | 1.450 | 1 | 0.229 | k = 1.150 p_0_ = 0.719 p_1_ = 0.126 p_2a_ = 0.132 p_2b_ = 0.023 ω_0 =_ 0.044 ω_1_ = 1.000 ω_2_ = 1.000 |  |
|  | branch 3 |  |  |  |  |  |  |  |
|  | A: two ratio | 63310.102 | A VS M0 | 10.075 | 1 | 0.002 | k = 1.071 ω_0 =_ 0.040 ω_1_ = 999.000 |  |
|  | B: two ratio fix ω=1 | 63310.402 | A VS B | 0.601 | 1 | 0.438 | k = 1.071 ω_0 =_ 0.040 ω_1_ = 1.000 |  |
|  | MA | 62773.888 |  |  |  |  | k = 1.152 p_0_ = 0.661 p_1_ = 0.116 p_2a_ = 0.190 p_2b_ = 0.033 ω_0 =_ 0.044 ω_1_ = 1.000 ω_2_ = 999.000 | 74 (0.975) |
|  | MA0 | 62774.780 | MA VS MA0 | 1.784 | 1 | 0.182 | k = 1.152 p_0_ = 0.634 p_1_ = 0.111 p_2a_ = 0.217 p_2b_ = 0.038 ω_0 =_ 0.044 ω_1_ = 1.000 ω_2_ = 1.000 |  |
| nd3 | M0: one ratio | 18832.555 |  |  |  |  | k = 1.194 ω = 0.030 |  |
|  | branch 2 |  |  |  |  |  |  |  |
|  | A: two ratio | 18831.132 | A VS M0 | 2.846 | 1 | 0.092 | k = 1.193 ω_0 =_ 0.029 ω_1_ = 999.000 |  |
|  | B: two ratio fix ω=1 | 18831.177 | A VS B | 0.090 | 1 | 0.764 | k = 1.193 ω_0 =_ 0.029 ω_1_ = 1.000 |  |
|  | MA | 18681.382 |  |  |  |  | k = 1.207 p_0_ = 0.818 p_1_ = 0.094 p_2a_ = 0.079 p_2b_ = 0.009 ω_0 =_ 0.027 ω_1_ = 1.000 ω_2_ = 999.000 |  |
|  | MA0 | 18682.149 | MA VS MA0 | 1.534 | 1 | 0.216 | k = 1.210 p_0_ = 0.795 p_1_ = 0.091 p_2a_ = 0.102 p_2b_ = 0.0112 ω_0 =_ 0.027 ω_1_ = 1.000 ω_2_ = 1.000 |  |
|  | branch 3 |  |  |  |  |  |  |  |
|  | A: two ratio | 18832.480 | A VS M0 | 0.150 | 1 | 0.698 | k = 1.195 ω_0 =_ 0.030 ω_1_ = 290.661 |  |
|  | B: two ratio fix ω=1 | 18832.485 | A VS B | 0.011 | 1 | 0.916 | k = 1.195 ω_0 =_ 0.030 ω_1_ = 1.000 |  |
|  | MA | 18685.014 |  |  |  |  | k = 1.214 p_0_ = 0.887 p_1_ = 0.102 p_2a_ = 0.010 p_2b_ = 0.001 ω_0 =_ 0.027 ω_1_ = 1.000 ω_2_ = 1.000 |  |
|  | MA0 | 18685.014 | MA VS MA0 | 0 | 1 | 1 | k = 1.214 p_0_ = 0.892 p_1_ = 0.102 p_2a_ = 0.005 p_2b_ = 0.001 ω_0 =_ 0.027 ω_1_ = 1.000 ω_2_ = 1.000 |  |
| nd4 | M0: one ratio | 69771.825 |  |  |  |  | k = 1.005 ω = 0.033 |  |
|  | branch 2 |  |  |  |  |  |  |  |
|  | A: two ratio | 69766.087 | A VS M0 | 11.478 | 1 | 0.001 | k = 1.005 ω_0 =_ 0.033 ω_1_ = 999.000 |  |
|  | B: two ratio fix ω=1 | 69766.294 | A VS B | 0.421 | 1 | 0.516 | k = 1.005 ω_0 =_ 0.033 ω_1_ = 1.000 |  |
|  | MA | 69125.348 |  |  |  |  | k = 0.972 p_0_ = 0.821 p_1_ = 0.078 p_2a_ = 0.092 p_2b_ = 0.009 ω_0 =_ 0.035 ω_1_ = 1.000 ω_2_ = 999.000 | 186 (0.907) |
|  | MA0 | 69127.742 | MA VS MA0 | 4.788 | 1 | 0.0286 | k = 0.972 p_0_ = 0.815 p_1_ = 0.077 p_2a_ = 0.099 p_2b_ = 0.009 ω_0 =_ 0.035 ω_1_ = 1.000 ω_2_ = 1.000 |  |
|  | branch 3 |  |  |  |  |  |  |  |
|  | A: two ratio | 69767.585 | A VS M0 | 8.481 | 1 | 0.004 | k = 1.007 ω_0 =_ 0.033 ω_1_ = 520.490 |  |
|  | B: two ratio fix ω=1 | 69767.642 | A VS B | 0.114 | 1 | 0.735 | k = 1.007 ω_0 =_ 0.033 ω_1_ = 1.000 |  |
|  | MA | 69121.836 |  |  |  |  | k = 0.974 p_0_ = 0.877 p_1_ = 0.083 p_2a_ = 0.036 p_2b_ = 0.003 ω_0 =_ 0.035 ω_1_ = 1.000 ω_2_ = 21.114 | 30 (0.961) 78 (0.962) 119 (0.997) 180 (0.986) 380 (0.995) 383 (0.929) |
|  | MA0 | 69127.578 | MA VS MA0 | 11.484 | 1 | 0.0007 | k = 0.972 p_0_ = 0.851 p_1_ = 0.081 p_2a_ = 0.062 p_2b_ = 0.006 ω_0 =_ 0.035 ω_1_ = 1.000 ω_2_ = 1.000 |  |
| nd4l | M0: one ratio | 15956.655 |  |  |  |  | k = 1.257 ω = 0.033 |  |
|  | branch 2 |  |  |  |  |  |  |  |
|  | A: two ratio | 15955.955 | A VS M0 | 1.400 | 1 | 0.237 | k = 1.259 ω_0 =_ 0.032 ω_1_ = 0.585 |  |
|  | B: two ratio fix ω=1 | 15955.956 | A VS B | 0.003 | 1 | 0.956 | k = 1.259 ω_0 =_ 0.032 ω_1_ = 1.000 |  |
|  | MA | 15790.873 |  |  |  |  | k = 1.540 p_0_ = 0.680 p_1_ = 0.181 p_2a_ = 0.109 p_2b_ = 0.029 ω_0 =_ 0.051 ω_1_ = 1.000 ω_2_ = 1.000 | 33 (0.922) |
|  | MA0 | 15792.136 | MA VS MA0 | 2.526 | 1 | 0.112 | k = 1.540 p_0_ = 0.806 p_1_ = 0.155 p_2a_ = 0.033 p_2b_ = 0.006 ω_0 =_ 0.044 ω_1_ = 1.000 ω_2_ = 1.000 |  |
|  | branch 3 |  |  |  |  |  |  |  |
|  | A: two ratio | 15955.604 | A VS M0 | 2.101 | 1 | 0.147 | k = 1.259 ω_0 =_ 0.032 ω_1_ = 999.000 |  |
|  | B: two ratio fix ω=1 | 15955.649 | A VS B | 0.089 | 1 | 0.765 | k = 1.259 ω_0 =_ 0.032 ω_1_ = 1.000 |  |
|  | MA | 15789.367 |  |  |  |  | k = 1.547 p_0_ = 0.734 p_1_ = 0.195 p_2a_ = 0.056 p_2b_ = 0.015 ω_0 =_ 0.050 ω_1_ = 1.000 ω_2_ = 999.000 | 24 (0.969) |
|  | MA0 | 15790.160 | MA VS MA0 | 1.586 | 1 | 0.208 | k = 1.544 p_0_ = 0.721 p_1_ = 0.192 p_2a_ = 0.069 p_2b_ = 0.018 ω_0 =_ 0.050 ω_1_ = 1.000 ω_2_ = 1.000 |  |
| nd5 | M0: one ratio | 90180.389 |  |  |  |  | k = 1.048 ω = 0.027 |  |
|  | branch 2 |  |  |  |  |  |  |  |
|  | A: two ratio | 90175.002 | A VS M0 | 10.773 | 1 | 0.001 | k = 1.048 ω_0 =_ 0.027 ω_1_ = 999.000 |  |
|  | B: two ratio fix ω=1 | 90175.212 | A VS B | 0.420 | 1 | 0.517 | k = 1.048 ω_0 =_ 0.027 ω_1_ = 1.000 |  |
|  | MA | 89252.419 |  |  |  |  | k = 1.312 p_0_ = 0.812 p_1_ = 0.188 p_2a_ = 0.000 p_2b_ = 0.000 ω_0 =_ 0.050 ω_1_ = 1.000 ω_2_ = 1.000 | 155 (0.897) 253 (0.873) |
|  | MA0 | 89249.384 | MA VS MA0 | 6.072 | 1 | 0.0137 | k = 1.313 p_0_ = 0.715 p_1_ = 0.165 p_2a_ = 0.098 p_2b_ = 0.023 ω_0 =_ 0.049 ω_1_ = 1.000 ω_2_ = 1.000 |  |
|  | branch 3 |  |  |  |  |  |  |  |
|  | A: two ratio | 90176.419 | A VS M0 | 7.940 | 1 | 0.005 | k = 1.048 ω_0 =_ 0.027 ω_1_ = 958.796 |  |
|  | B: two ratio fix ω=1 | 90176.564 | A VS B | 0.291 | 1 | 0.590 | k = 1.048 ω_0 =_ 0.027 ω_1_ = 1.000 |  |
|  | MA | 89252.419 |  |  |  |  | k = 1.312 p_0_ = 0.812 p_1_ = 0.188 p_2a_ = 0.000 p_2b_ = 0.000 ω_0 =_ 0.050 ω_1_ = 1.000 ω_2_ = 1.000 |  |
|  | MA0 | 89252.419 | MA VS MA0 | 0 | 1 | 1 | k = 1.312 p_0_ = 0.812 p_1_ = 0.188 p_2a_ = 0.000 p_2b_ = 0.000 ω_0 =_ 0.050 ω_1_ = 1.000 ω_2_ = 1.000 |  |
| nd6 | M0: one ratio | 32896.124 |  |  |  |  | k = 1.089 ω = 0.025 |  |
|  | branch 2 |  |  |  |  |  |  |  |
|  | A: two ratio | 32894.793 | A VS M0 | 2.663 | 1 | 0.103 | k = 1.090 ω_0 =_ 0.024 ω_1_ = 999.000 |  |
|  | B: two ratio fix ω=1 | 32894.845 | A VS B | 0.105 | 1 | 0.746 | k = 1.090 ω_0 =_ 0.024 ω_1_ = 1.000 |  |
|  | MA | 32385.329 |  |  |  |  | k = 1.563 p_0_ = 0.510 p_1_ = 0.372 p_2a_ = 0.068 p_2b_ = 0.050 ω_0 =_ 0.074 ω_1_ = 1.000 ω_2_ = 1.000 |  |
|  | MA0 | 32385.329 | MA VS MA0 | 0 | 1 | 1 | k = 1.563 p_0_ = 0.510 p_1_ = 0.372 p_2a_ = 0.068 p_2b_ = 0.050 ω_0 =_ 0.074 ω_1_ = 1.000 ω_2_ = 1.000 |  |
|  | branch 3 |  |  |  |  |  |  |  |
|  | A: two ratio | 32895.779 | A VS M0 | 0.691 | 1 | 0.406 | k = 1.090 ω_0 =_ 0.024 ω_1_ = 404.709 |  |
|  | B: two ratio fix ω=1 | 32895.789 | A VS B | 0.021 | 1 | 0.885 | k = 1.090 ω_0 =_ 0.024 ω_1_ = 1.000 |  |
|  | MA | 32385.232 |  |  |  |  | k = 1.565 p_0_ = 0.450 p_1_ = 0.329 p_2a_ = 0.127 p_2b_ = 0.093 ω_0 =_ 0.074 ω_1_ = 1.000 ω_2_ = 1.000 | 171 (0.803) |
|  | MA0 | 32385.232 | MA VS MA0 | 0 | 1 | 1 | k = 1.565 p_0_ = 0.450 p_1_ = 0.329 p_2a_ = 0.127 p_2b_ = 0.093 ω_0 =_ 0.074 ω_1_ = 1.000 ω_2_ = 1.000 |  |

*: branch 2 and branch 3 are showed in Figure 1 and represent LAC of Pterygota and LAC of Neoptera, respectively.
