# Supplementary material for: Adaptive Evolution of Mitochondrial Energy Metabolism Genes Associated with Increased Energy Demand in Flying Insects
Source: PLoS One. 2014 Jun 11;9(6):e99120. doi: 10.1371/journal.pone.0099120 (PMC4053383; doi:10.1371/journal.pone.0099120)
Supplement: Table S3 — Evidence of positive selection for mtDNA genes in insect orders with branch site model. (DOC) [file pone.0099120.s004.doc]

**Table S3 Evidence of positive selection for mtDNA genes of insect orders with branch site model.**

| Gene | Order* | Model | -ln L | 2ΔlnL | P value | ω values | Number of Positively selected site(BEB:P>95%) |
| --- | --- | --- | --- | --- | --- | --- | --- |
| atp6 |  |  |  |  |  |  |  |
|  | p | MA | 31530.957 |  |  | ω0 = 0.033, ω1 = 1, ω2 = 999 | 3 |
|  |  | MA0 | 31537.163 | 12.412 | 0.0004 | ω0 = 0.033, ω1 = 1, ω2 = 1 |  |
|  | l | MA | 31541.121 |  |  | ω0 = 0.033, ω1 = 1, ω2 = 1 |  |
|  |  | MA0 | 31544.627 | 7.011 | 0.0081 | ω0 = 0.033, ω1 = 1, ω2 = 1 |  |
|  | q | MA | 31530.707 |  |  | ω0 = 0.033, ω1 = 1, ω2 = 999 |  |
|  |  | MA0 | 31532.824 | 4.234 | 0.0396 | ω0 = 0.033, ω1 = 1, ω2 = 1 |  |
| atp8 |  |  |  |  |  |  |  |
|  | i | MA | 9698.317 |  |  | ω0 = 0.082, ω1 = 1, ω2 = 999 | 5 |
|  |  | MA0 | 9700.322 | 4.011 | 0.045 | ω0 = 0.082, ω1 = 1, ω2 = 1 |  |
| cox1 |  |  |  |  |  |  |  |
|  | n | MA | 57538.214 |  |  | ω0 = 0.021, ω1 = 1, ω2 = 10.981 | 7 |
|  |  | MA0 | 57543.847 | 11.265 | 0.0008 | ω0 = 0.021, ω1 = 1, ω2 = 1 |  |
|  | l | MA | 57530.591 |  |  | ω0 = 0.021, ω1 = 1, ω2 = 999 |  |
|  |  | MA0 | 57536.131 | 11.080 | 0.0009 | ω0 = 0.021, ω1 = 1, ω2 = 1 |  |
|  | m | MA | 57557.662 |  |  | ω0 = 0.021, ω1 = 1, ω2 = 999 | 2 |
|  |  | MA0 | 57560.133 | 4.943 | 0.0262 | ω0 = 0.021, ω1 = 1, ω2 = 1 |  |
|  | r | MA | 57522.725 |  |  | ω0 = 0.021, ω1 = 1, ω2 = 2.810 | 7 |
|  |  | MA0 | 57524.929 | 4.408 | 0.0358 | ω0 = 0.021, ω1 = 1, ω2 = 1 |  |
|  | q | MA | 57539.641 |  |  | ω0 = 0.021, ω1 = 1, ω2 = 999 |  |
|  |  | MA0 | 57541.610 | 3.936 | 0.0473 | ω0 = 0.021, ω1 = 1, ω2 = 1 |  |
| cox2 |  |  |  |  |  |  |  |
|  | m | MA | 29853.017 |  |  | ω0 = 0.026, ω1 = 1, ω2 = 999 |  |
|  |  | MA0 | 29855.836 | 5.639 | 0.0176 | ω0 = 0.027, ω1 = 1, ω2 = 1 |  |
|  | f | MA | 29851.973 |  |  | ω0 = 0.027, ω1 = 1, ω2 = 999 |  |
|  |  | MA0 | 29854.494 | 5.042 | 0.0247 | ω0 = 0.027, ω1 = 1, ω2 = 1 |  |
|  | d | MA | 29850.736 |  |  | ω0 = 0.027, ω1 = 1, ω2 = 999 |  |
|  |  | MA0 | 29852.673 | 3.874 | 0.0490 | ω0 = 0.027, ω1 = 1, ω2 = 1 |  |
| cox3 |  |  |  |  |  |  |  |
|  | j | MA | 35571.051 |  |  | ω0 = 0.040, ω1 = 1, ω2 = 14.318 | 34 |
|  |  | MA0 | 35580.606 | 19.109 | 0 | ω0 = 0.041, ω1 = 1, ω2 = 1 |  |
|  | i | MA | 35606.997 |  |  | ω0 = 0.042, ω1 = 1, ω2 = 999 | 1 |
|  |  | MA0 | 35613.333 | 12.672 | 0.0004 | ω0 = 0.042, ω1 = 1, ω2 = 1 |  |
|  | a | MA | 35612.530 |  |  | ω0 = 0.042, ω1 = 1, ω2 = 25.724 | 2 |
|  |  | MA0 | 35615.448 | 5.837 | 0.0157 | ω0 = 0.042, ω1 = 1, ω2 = 1 |  |
|  | r | MA | 35597.337 |  |  | ω0 = 0.041, ω1 = 1, ω2 = 4.173 | 7 |
|  |  | MA0 | 35599.914 | 5.154 | 0.0232 | ω0 = 0.041, ω1 = 1, ω2 = 1 |  |
|  | q | MA | 35603.156 |  |  | ω0 = 0.040, ω1 = 1, ω2 = 999 | 1 |
|  |  | MA0 | 35605.647 | 4.982 | 0.0256 | ω0 = 0.040, ω1 = 1, ω2 = 1 |  |
|  | t | MA | 35607.923 |  |  | ω0 = 0.042, ω1 = 1, ω2 = 49.416 | 3 |
|  |  | MA0 | 35610.129 | 4.411 | 0.0357 | ω0 = 0.042, ω1 = 1, ω2 = 1 |  |
| cytb |  |  |  |  |  |  |  |
|  | m | MA | 49600.746 |  |  | ω0 = 0.034, ω1 = 1, ω2 = 999 | 2 |
|  |  | MA0 | 49606.113 | 10.735 | 0.0011 | ω0 = 0.034, ω1 = 1, ω2 =1 |  |
|  | n | MA | 49592.498 |  |  | ω0 = 0.034, ω1 = 1, ω2 = 8.335 | 5 |
|  |  | MA0 | 49597.698 | 10.400 | 0.0013 | ω0 = 0.034, ω1 = 1, ω2 =1 |  |
|  | l | MA | 49587.277 |  |  | ω0 = 0.034, ω1 = 1, ω2 = 999 |  |
|  |  | MA0 | 49591.096 | 7.637 | 0.0057 | ω0 = 0.034, ω1 = 1, ω2 =1 |  |
|  | q | MA | 49589.852 |  |  | ω0 = 0.034, ω1 = 1, ω2 =248.161 | 14 |
|  |  | MA0 | 49592.609 | 5.513 | 0.0189 | ω0 = 0.034, ω1 = 1, ω2 =1 |  |
|  | d | MA | 49601.786 |  |  | ω0 = 0.034, ω1 = 1, ω2 =8.339 | 2 |
|  |  | MA0 | 49604.418 | 5.264 | 0.0218 | ω0 = 0.034, ω1 = 1, ω2 =1 |  |
| nd1 |  |  |  |  |  |  |  |
|  | p | MA | 44472.574 |  |  | ω0 = 0.030, ω1 = 1, ω2 =999 | 2 |
|  |  | MA0 | 44479.698 | 14.249 | 0.0002 | ω0 = 0.030, ω1 = 1, ω2 =1 |  |
|  | i | MA | 44480.933 |  |  | ω0 = 0.030, ω1 = 1, ω2 =999 | 3 |
|  |  | MA0 | 44483.756 | 5.646 | 0.0175 | ω0 = 0.030, ω1 = 1, ω2 =1 |  |
|  | q | MA | 44470.495 |  |  | ω0 = 0.030, ω1 = 1, ω2 =999 |  |
|  |  | MA0 | 44472.745 | 4.500 | 0.0339 | ω0 = 0.030, ω1 = 1, ω2 =1 |  |
|  | s | MA | 44473.712 |  |  | ω0 = 0.030, ω1 = 1, ω2 =999 | 1 |
|  |  | MA0 | 44480.127 | 12.830 | 0.0003 | ω0 = 0.030, ω1 = 1, ω2 =1 |  |
| nd2 |  |  |  |  |  |  |  |
|  | i | MA | 62772.647 |  |  | ω0 = 0.044, ω1 = 1, ω2 =999 |  |
|  |  | MA0 | 62776.370 | 7.445 | 0.0064 | ω0 = 0.044, ω1 = 1, ω2 =1 |  |
|  | l | MA | 62768.851 |  |  | ω0 = 0.044, ω1 = 1, ω2 =999 | 2 |
|  |  | MA0 | 62771.842 | 5.982 | 0.0144 | ω0 = 0.044, ω1 = 1, ω2 =1 |  |
|  | q | MA | 62763.941 |  |  | ω0 = 0.045, ω1 = 1, ω2 =999 |  |
|  |  | MA0 | 62765.924 | 3.967 | 0.0464 | ω0 = 0.044, ω1 = 1, ω2 =1 |  |
| nd4 |  |  |  |  |  |  |  |
|  | q | MA | 69115.157 |  |  | ω0 = 0.035, ω1 = 1, ω2 =999 |  |
|  |  | MA0 | 69118.176 | 6.038 | 0.0140 | ω0 = 0.035, ω1 = 1, ω2 =1 |  |
|  | s | MA | 69133.136 |  |  | ω0 = 0.035, ω1 = 1, ω2 =135.889 |  |
|  |  | MA0 | 69136.021 | 5.770 | 0.0163 | ω0 = 0.035, ω1 = 1, ω2 =1 |  |
|  | b | MA | 69110.100 |  |  | ω0 = 0.035, ω1 = 1, ω2 =4.719 |  |
|  |  | MA0 | 69112.397 | 4.594 | 0.0321 | ω0 = 0.035, ω1 = 1, ω2 =1 |  |
| nd4l |  |  |  |  |  |  |  |
|  | l | MA | 89218.418 |  |  | ω0 = 0.050, ω1 = 1, ω2 =999 | 4 |
|  |  | MA0 | 89230.673 | 24.510 | 0.0000 | ω0 = 0.049, ω1 = 1, ω2 =1 |  |
| nd5 |  |  |  |  |  |  |  |
|  | q | MA | 89222.194 |  |  | ω0 = 0.049, ω1 = 1, ω2 =999 | 6 |
|  |  | MA0 | 89229.419 | 14.451 | 0.0001 | ω0 = 0.049, ω1 = 1, ω2 =1 |  |
|  | n | MA | 89228.789 |  |  | ω0 = 0.049, ω1 = 1, ω2 =998.992 | 6 |
|  |  | MA0 | 89235.708 | 13.838 | 0.0002 | ω0 = 0.049, ω1 = 1, ω2 =1 |  |
|  | i | MA | 89244.824 |  |  | ω0 = 0.049, ω1 = 1, ω2 =999 |  |
|  |  | MA0 | 89250.716 | 11.783 | 0.0006 | ω0 = 0.049, ω1 = 1, ω2 =1 |  |
|  | m | MA | 89238.188 |  |  | ω0 = 0.049, ω1 = 1, ω2 =999 | 8 |
|  |  | MA0 | 89243.028 | 9.680 | 0.0019 | ω0 = 0.049, ω1 = 1, ω2 =1 |  |
|  | p | MA | 89248.413 |  |  | ω0 = 0.049, ω1 = 1, ω2 =999 | 2 |
|  |  | MA0 | 89251.483 | 6.139 | 0.0132 | ω0 = 0.049, ω1 = 1, ω2 =1 |  |
|  | r | MA | 89214.776 |  |  | ω0 = 0.051, ω1 = 1, ω2 =22.366 | 20 |
|  |  | MA0 | 89217.841 | 6.130 | 0.0133 | ω0 = 0.051, ω1 = 1, ω2 =1 |  |
|  | i | MA | 89224.919 |  |  | ω0 = 0.049, ω1 = 1, ω2 =12.490 | 13 |
|  |  | MA0 | 89227.321 | 4.805 | 0.0284 | ω0 = 0.049, ω1 = 1, ω2 =1 |  |
|  | s | MA | 89244.255 |  |  | ω0 = 0.049, ω1 = 1, ω2 =47.810 | 2 |
|  |  | MA0 | 89246.649 | 4.788 | 0.0287 | ω0 = 0.049, ω1 = 1, ω2 =1 |  |
| nd6 |  |  |  |  |  |  |  |
|  | m | MA | 32377.177 |  |  | ω0 = 0.072, ω1 = 1, ω2 =999 |  |
|  |  | MA0 | 32382.590 | 10.827 | 0.0010 | ω0 = 0.074, ω1 = 1, ω2 =1 | 4 |
|  | s | MA | 32380.711 |  |  | ω0 = 0.074, ω1 = 1, ω2 =139.165 | 1 |
|  |  | MA0 | 32384.215 | 7.009 | 0.0081 | ω0 = 0.074, ω1 = 1, ω2 =1 |  |
|  | d | MA | 32380.550 |  |  | ω0 = 0.074, ω1 = 1, ω2 =999 | 1 |
|  |  | MA0 | 32383.400 | 5.699 | 0.0170 | ω0 = 0.074, ω1 = 1, ω2 =1 |  |
|  | n | MA | 32377.061 |  |  | ω0 = 0.074, ω1 = 1, ω2 =11.990 |  |
|  |  | MA0 | 32379.776 | 5.431 | 0.0198 | ω0 = 0.073, ω1 = 1, ω2 =1 | 3 |
|  | l | MA | 32381.329 |  |  | ω0 = 0.074, ω1 = 1, ω2 =999 | 12 |
|  |  | MA0 | 32383.502 | 4.347 | 0.0371 | ω0 = 0.074, ω1 = 1, ω2 =1 |  |

*: The letters in order column represent orders showed in Table S1
