# Supplementary material for: Adaptive Evolution of Mitochondrial Energy Metabolism Genes Associated with Increased Energy Demand in Flying Insects
Source: PLoS One. 2014 Jun 11;9(6):e99120. doi: 10.1371/journal.pone.0099120 (PMC4053383; doi:10.1371/journal.pone.0099120)
Supplement: Table S4 — Evidence of positive selection for mtDNA genes of each ancestor branch with branch site model. (DOC) [file pone.0099120.s005.doc]

**Table S4 Evidence of positive selection for mtDNA genes of each ancestor branch with branch site model**

| Gene | branch* | Model | -ln L | 2ΔlnL | P value | ω values | Number of Positively selected site (BEB: P>95%) |
| --- | --- | --- | --- | --- | --- | --- | --- |
| atp6 |  |  |  |  |  |  |  |
|  | 12 | MA | 31532.930 |  |  | ω0 = 0.033, ω1 = 1, ω2 = 999 | 1 |
|  |  | MA0 | 31538.657 | 11.454 | 0.0007 | ω0 = 0.033, ω1 = 1, ω2 = 1 |  |
| atp8 |  |  |  |  |  |  |  |
|  | 14 | MA | 9699.785 |  |  | ω0 = 0.083, ω1 = 1, ω2 = 999 |  |
|  |  | MA0 | 9702.752 | 5.933 | 0.015 | ω0 = 0.083, ω1 = 1, ω2 = 1 |  |
| cox1 |  |  |  |  |  |  |  |
|  | 14 | MA | 57550.064 |  |  | ω0 = 0.021, ω1 = 1, ω2 = 999 |  |
|  |  | MA0 | 57556.519 | 12.910 | 0.0003 | ω0 = 0.021, ω1 = 1, ω2 = 1 |  |
|  | 10 | MA | 57553.132 |  |  | ω0 = 0.021, ω1 = 1, ω2 = 999 | 1 |
|  |  | MA0 | 57559.398 | 12.531 | 0.0004 | ω0 = 0.021, ω1 = 1, ω2 = 1 |  |
|  | 12 | MA | 57557.483 |  |  | ω0 = 0.021, ω1 = 1, ω2 = 999 |  |
|  |  | MA0 | 57561.668 | 8.371 | 0.004 | ω0 = 0.021, ω1 = 1, ω2 = 1 |  |
|  | 13 | MA | 57543.492 |  |  | ω0 = 0.021, ω1 = 1, ω2 = 999 |  |
|  |  | MA0 | 57547.594 | 8.204 | 0.004 | ω0 = 0.021, ω1 = 1, ω2 = 1 |  |
|  | 8 | MA | 57553.788 |  |  | ω0 = 0.021, ω1 = 1, ω2 = 999 |  |
|  |  | MA0 | 57557.875 | 8.173 | 0.004 | ω0 = 0.021, ω1 = 1, ω2 = 1 |  |
|  | 3 | MA | 57551.929 |  |  | ω0 = 0.021, ω1 = 1, ω2 = 999 | 2 |
|  |  | MA0 | 57555.613 | 7.367 | 0.007 | ω0 = 0.021, ω1 = 1, ω2 = 1 |  |
|  | 5 | MA | 57555.274 |  |  | ω0 = 0.021, ω1 = 1, ω2 = 22.055 | 3 |
|  |  | MA0 | 57558.689 | 6.830 | 0.009 | ω0 = 0.021, ω1 = 1, ω2 = 1 |  |
|  | 17 | MA | 57557.776 |  |  | ω0 = 0.021, ω1 = 1, ω2 = 999 |  |
|  |  | MA0 | 57560.020 | 4.489 | 0.034 | ω0 = 0.021, ω1 = 1, ω2 = 1 |  |
| cox2 |  |  |  |  |  |  |  |
|  | 9 | MA | 29847.384 |  |  | ω0 = 0.026, ω1 = 1, ω2 = 999 | 1 |
|  |  | MA0 | 29849.374 | 3.980 | 0.046 | ω0 = 0.027, ω1 = 1, ω2 = 1 |  |
|  | 16 | MA | 29846.936 |  |  | ω0 = 0.027, ω1 = 1, ω2 = 999 |  |
|  |  | MA0 | 29849.578 | 5.285 | 0.022 | ω0 = 0.027, ω1 = 1, ω2 = 1 |  |
|  | 1 | MA | 29845.442 |  |  | ω0 = 0.027, ω1 = 1, ω2 = 999 |  |
|  |  | MA0 | 29848.104 | 5.324 | 0.021 | ω0 = 0.027, ω1 = 1, ω2 = 1 |  |
|  | 14 | MA | 29841.840 |  |  | ω0 = 0.026, ω1 = 1, ω2 = 999 |  |
|  |  | MA0 | 29846.990 | 10.300 | 0.001 | ω0 = 0.026, ω1 = 1, ω2 = 1 |  |
| cox3 |  |  |  |  |  |  |  |
|  | 11 | MA | 35611.976 |  |  | ω0 = 0.040, ω1 = 1, ω2 = 24.658 | 2 |
|  |  | MA0 | 35614.049 | 4.144 | 0.042 | ω0 = 0.041, ω1 = 1, ω2 = 1 |  |
|  | 14 | MA | 35610.158 |  |  | ω0 = 0.042, ω1 = 1, ω2 = 999 |  |
|  |  | MA0 | 35615.303 | 10.290 | 0.001 | ω0 = 0.042, ω1 = 1, ω2 = 1 |  |
|  | 15 | MA | 35602.395 |  |  | ω0 = 0.042, ω1 = 1, ω2 = 999 | 3 |
|  |  | MA0 | 35608.632 | 12.474 | 0.0004 | ω0 = 0.042, ω1 = 1, ω2 = 1 |  |
| cytb |  |  |  |  |  |  |  |
|  | 13 | MA | 49598.244 |  |  | ω0 = 0.034, ω1 = 1, ω2 = 999 |  |
|  |  | MA0 | 49600.585 | 4.683 | 0.030 | ω0 = 0.034, ω1 = 1, ω2 =1 |  |
|  | 19 | MA | 49593.754 |  |  | ω0 = 0.034, ω1 = 1, ω2 = 49.637 | 4 |
|  |  | MA0 | 49600.202 | 12.895 | 0.0003 | ω0 = 0.034, ω1 = 1, ω2 =1 |  |
|  | 1 | MA | 49595.802 |  |  | ω0 = 0.034, ω1 = 1, ω2 = 999 |  |
|  |  | MA0 | 49600.360 | 9.116 | 0.003 | ω0 = 0.034, ω1 = 1, ω2 =1 |  |
|  | 18 | MA | 49597.986 |  |  | ω0 = 0.034, ω1 = 1, ω2 =999 |  |
|  |  | MA0 | 49602.143 | 8.313 | 0.004 | ω0 = 0.034, ω1 = 1, ω2 =1 |  |
|  | 8 | MA | 49606.207 |  |  | ω0 = 0.034, ω1 = 1, ω2 =999 |  |
|  |  | MA0 | 49608.881 | 5.347 | 0.021 | ω0 = 0.034, ω1 = 1, ω2 =1 |  |
|  | 7 | MA | 49606.749 |  |  | ω0 = 0.034, ω1 = 1, ω2 =999 |  |
|  |  | MA0 | 49609.419 | 5.339 | 0.021 | ω0 = 0.034, ω1 = 1, ω2 =1 |  |
|  | 12 | MA | 49607.706 |  |  | ω0 = 0.034, ω1 = 1, ω2 =999 |  |
|  |  | MA0 | 49609.982 | 4.551 | 0.033 | ω0 = 0.034, ω1 = 1, ω2 =1 |  |
|  | 11 | MA | 49595.357 |  |  | ω0 = 0.034, ω1 = 1, ω2 =999 |  |
|  |  | MA0 | 49603.872 | 17.031 | 0 | ω0 = 0.034, ω1 = 1, ω2 =1 |  |
|  | 14 | MA | 49601.059 |  |  | ω0 = 0.034, ω1 = 1, ω2 =999 |  |
|  |  | MA0 | 49605.503 | 8.888 | 0.003 | ω0 = 0.034, ω1 = 1, ω2 =1 |  |
| nd1 |  |  |  |  |  |  |  |
|  | 19 | MA | 44476.985 |  |  | ω0 = 0.030, ω1 = 1, ω2 =999 | 1 |
|  |  | MA0 | 44480.984 | 7.996 | 0.005 | ω0 = 0.030, ω1 = 1, ω2 =1 |  |
|  | 18 | MA | 44476.606 |  |  | ω0 = 0.030, ω1 = 1, ω2 =999 |  |
|  |  | MA0 | 44481.509 | 9.808 | 0.002 | ω0 = 0.030, ω1 = 1, ω2 =1 |  |
|  | 14 | MA | 44479.631 |  |  | ω0 = 0.030, ω1 = 1, ω2 =999 |  |
|  |  | MA0 | 44482.259 | 5.258 | 0.022 | ω0 = 0.030, ω1 = 1, ω2 =1 |  |
|  | 15 | MA | 44482.117 |  |  | ω0 = 0.030, ω1 = 1, ω2 =999 |  |
|  |  | MA0 | 44484.987 | 5.740 | 0.017 | ω0 = 0.030, ω1 = 1, ω2 =1 |  |
| nd2 |  |  |  |  |  |  |  |
|  | 1 | MA | 62768.871 |  |  | ω0 = 0.044, ω1 = 1, ω2 =999 |  |
|  |  | MA0 | 62770.885 | 4.028 | 0.045 | ω0 = 0.044, ω1 = 1, ω2 =1 |  |
|  | 18 | MA | 62774.579 |  |  | ω0 = 0.044, ω1 = 1, ω2 =999 |  |
|  |  | MA0 | 62777.538 | 5.918 | 0.015 | ω0 = 0.044, ω1 = 1, ω2 =1 |  |
|  | 11 | MA | 62769.606 |  |  | ω0 = 0.045, ω1 = 1, ω2 =999 | 2 |
|  |  | MA0 | 62772.095 | 4.977 | 0.026 | ω0 = 0.044, ω1 = 1, ω2 =1 |  |
|  | 10 | MA | 62771.496 |  |  | ω0 = 0.045, ω1 = 1, ω2 =999 | 1 |
|  |  | MA0 | 62775.503 | 8.013 | 0.005 | ω0 = 0.044, ω1 = 1, ω2 =1 |  |
|  | 15 | MA | 62768.002 |  |  | ω0 = 0.045, ω1 = 1, ω2 =999 | 2 |
|  |  | MA0 | 62771.770 | 7.536 | 0.006 | ω0 = 0.044, ω1 = 1, ω2 =1 |  |
| nd3 |  |  |  |  |  |  |  |
|  | 19 | MA | 18676.636 |  |  | ω0 = 0.026, ω1 = 1, ω2 =999 |  |
|  |  | MA0 | 18679.069 | 4.866 | 0.027 | ω0 = 0.027, ω1 = 1, ω2 =1 |  |
|  | 17 | MA | 18684.564 |  |  | ω0 = 0.026, ω1 = 1, ω2 =184.093 |  |
|  |  | MA0 | 18853.566 | 338.002 | 0 | ω0 = 0.027, ω1 = 1, ω2 =1 |  |
| nd4 |  |  |  |  |  |  |  |
|  | 13 | MA | 69123.830 |  |  | ω0 = 0.035, ω1 = 1, ω2 =999 |  |
|  |  | MA0 | 69126.182 | 4.703 | 0.030 | ω0 = 0.035, ω1 = 1, ω2 =1 |  |
|  | 19 | MA | 69124.227 |  |  | ω0 = 0.035, ω1 = 1, ω2 =999 | 1 |
|  |  | MA0 | 69127.922 | 7.391 | 0.007 | ω0 = 0.035, ω1 = 1, ω2 =1 |  |
|  | 4 | MA | 69133.554 |  |  | ω0 = 0.035, ω1 = 1, ω2 =30.974 |  |
|  |  | MA0 | 69135.740 | 4.372 | 0.037 | ω0 = 0.035, ω1 = 1, ω2 =1 |  |
|  | 17 | MA | 69129.125 |  |  | ω0 = 0.035, ω1 = 1, ω2 =999 |  |
|  |  | MA0 | 69133.014 | 7.778 | 0.005 | ω0 = 0.035, ω1 = 1, ω2 =1 |  |
|  | 1 | MA | 69111.537 |  |  | ω0 = 0.035, ω1 = 1, ω2 =999 |  |
|  |  | MA0 | 69117.211 | 11.347 | 0.001 | ω0 = 0.035, ω1 = 1, ω2 =1 |  |
|  | 11 | MA | 69112.414 |  |  | ω0 = 0.035, ω1 = 1, ω2 =999 | 2 |
|  |  | MA0 | 69128.388 | 31.947 | 0 | ω0 = 0.035, ω1 = 1, ω2 =1 |  |
|  | 10 | MA | 69127.743 |  |  | ω0 = 0.035, ω1 = 1, ω2 =999 |  |
|  |  | MA0 | 69131.311 | 7.136 | 0.008 | ω0 = 0.035, ω1 = 1, ω2 =1 |  |
|  | 14 | MA | 69127.885 |  |  | ω0 = 0.035, ω1 = 1, ω2 =999 |  |
|  |  | MA0 | 69131.433 | 7.094 | 0.007 | ω0 = 0.035, ω1 = 1, ω2 =1 |  |
|  | 15 | MA | 69124.532 |  |  | ω0 = 0.035, ω1 = 1, ω2 =999 |  |
|  |  | MA0 | 69127.557 | 6.050 | 0.014 | ω0 = 0.035, ω1 = 1, ω2 =1 |  |
| nd4l |  |  |  |  |  |  |  |
|  | 8 | MA | 15786.130 |  |  | ω0 = 0.050, ω1 = 1, ω2 =999 | 1 |
|  |  | MA0 | 15791.668 | 11.076 | 0.001 | ω0 = 0.050, ω1 = 1, ω2 =1 |  |
|  | 7 | MA | 15786.920 |  |  | ω0 = 0.050, ω1 = 1, ω2 =999 | 1 |
|  |  | MA0 | 15790.690 | 7.540 | 0.006 | ω0 = 0.050, ω1 = 1, ω2 =1 |  |
|  | 11 | MA | 15784.505 |  |  | ω0 = 0.050, ω1 = 1, ω2 =999 | 2 |
|  |  | MA0 | 15788.124 | 7.238 | 0.007 | ω0 = 0.050, ω1 = 1, ω2 =1 |  |
|  | 10 | MA | 15782.954 |  |  | ω0 = 0.050, ω1 = 1, ω2 =145.892 | 4 |
|  |  | MA0 | 15787.649 | 9.391 | 0.002 | ω0 = 0.050, ω1 = 1, ω2 =1 |  |
| nd5 |  |  |  |  |  |  |  |
|  | 13 | MA | 89236.928 |  |  | ω0 = 0.049, ω1 = 1, ω2 =999 | 5 |
|  |  | MA0 | 89240.492 | 7.126 | 0.007 | ω0 = 0.049, ω1 = 1, ω2 =1 |  |
|  | 4 | MA | 89248.757 |  |  | ω0 = 0.049, ω1 = 1, ω2 =999 | 1 |
|  |  | MA0 | 89251.228 | 4.941 | 0.026 | ω0 = 0.049, ω1 = 1, ω2 =1 |  |
|  | 16 | MA | 89247.063 |  |  | ω0 = 0.049, ω1 = 1, ω2 =999 |  |
|  |  | MA0 | 89249.237 | 4.347 | 0.037 | ω0 = 0.049, ω1 = 1, ω2 =1 |  |
|  | 1 | MA | 89234.521 |  |  | ω0 = 0.049, ω1 = 1, ω2 =5.708 | 6 |
|  |  | MA0 | 89237.809 | 6.576 | 0.010 | ω0 = 0.049, ω1 = 1, ω2 =1 |  |
|  | 12 | MA | 89240.079 |  |  | ω0 = 0.049, ω1 = 1, ω2 =999 | 1 |
|  |  | MA0 | 89249.332 | 18.506 | 0 | ω0 = 0.049, ω1 = 1, ω2 =1 |  |
|  | 11 | MA | 89241.848 |  |  | ω0 = 0.051, ω1 = 1, ω2 =28.136 | 3 |
|  |  | MA0 | 89245.391 | 7.086 | 0.008 | ω0 = 0.051, ω1 = 1, ω2 =1 |  |
|  | 14 | MA | 89243.092 |  |  | ω0 = 0.049, ω1 = 1, ω2 =999 |  |
|  |  | MA0 | 89249.351 | 12.518 | 0 | ω0 = 0.049, ω1 = 1, ω2 =1 |  |
|  | 15 | MA | 89236.801 |  |  | ω0 = 0.049, ω1 = 1, ω2 =999 | 1 |
|  |  | MA0 | 89241.795 | 9.988 | 0.001 | ω0 = 0.049, ω1 = 1, ω2 =1 |  |
| nd6 |  |  |  |  |  |  |  |
|  | 9 | MA | 32380.253 |  |  | ω0 = 0.074, ω1 = 1, ω2 =999 | 1 |
|  |  | MA0 | 32383.248 | 5.988 | 0.014 | ω0 = 0.074, ω1 = 1, ω2 =1 |  |
|  | 5 | MA | 32376.690 |  |  | ω0 = 0.073, ω1 = 1, ω2 =999 | 1 |
|  |  | MA0 | 32383.799 | 14.218 | 0.0001 | ω0 = 0.073, ω1 = 1, ω2 =1 |  |
|  | 1 | MA | 32378.530 |  |  | ω0 = 0.074, ω1 = 1, ω2 =999 | 3 |
|  |  | MA0 | 32382.529 | 7.999 | 0.004 | ω0 = 0.074, ω1 = 1, ω2 =1 |  |
|  | 8 | MA | 32380.356 |  |  | ω0 = 0.074, ω1 = 1, ω2 =999 |  |
|  |  | MA0 | 32384.950 | 9.189 | 0.002 | ω0 = 0.073, ω1 = 1, ω2 =1 |  |
|  | 11 | MA | 32375.290 |  |  | ω0 = 0.074, ω1 = 1, ω2 =999 | 4 |
|  |  | MA0 | 32380.917 | 11.255 | 0.001 | ω0 = 0.074, ω1 = 1, ω2 =1 |  |
|  | 10 | MA | 32379.278 |  |  | ω0 = 0.074, ω1 = 1, ω2 =999 | 1 |
|  |  | MA0 | 32382.792 | 7.028 | 0.008 | ω0 = 0.074, ω1 = 1, ω2 =1 |  |
|  | 14 | MA | 32382.112 |  |  | ω0 = 0.074, ω1 = 1, ω2 =999 | 1 |
|  |  | MA0 | 32384.264 | 4.303 | 0.038 | ω0 = 0.074, ω1 = 1, ω2 =1 |  |

*: The number represent the branch showed in Figure 2
